# Hydrogen Impact: A Review on Diffusibility, Embrittlement Mechanisms, and Characterization

**DOI:** 10.3390/ma17040965

**Published:** 2024-02-19

**Authors:** Qidong Li, Hesamedin Ghadiani, Vahid Jalilvand, Tahrim Alam, Zoheir Farhat, Md. Aminul Islam

**Affiliations:** 1 Department of Mechanical Engineering, Dalhousie University, Halifax, NS B3H 4R2, Canada; qidong98@student.ubc.ca (Q.L.); hs460468@dal.ca (H.G.);; 2 Enbridge Gas Inc., Ottawa, ON K1K 2C7, Canada; tahrim.alam@enbridge.com; 3 Mining Wear and Corrosion Laboratory, National Research Council Canada, Vancouver, BC V6T 1W5, Canada; mdaminul.islam@nrc-cnrc.gc.ca

**Keywords:** hydrogen embrittlement, hydrogen diffusion, damage mechanisms, mechanical properties

## Abstract

Hydrogen embrittlement (HE) is a broadly recognized phenomenon in metallic materials. If not well understood and managed, HE may lead to catastrophic environmental failures in vessels containing hydrogen, such as pipelines and storage tanks. HE can affect the mechanical properties of materials such as ductility, toughness, and strength, mainly through the interaction between metal defects and hydrogen. Various phenomena such as hydrogen adsorption, hydrogen diffusion, and hydrogen interactions with intrinsic trapping sites like dislocations, voids, grain boundaries, and oxide/matrix interfaces are involved in this process. It is important to understand HE mechanisms to develop effective hydrogen resistant strategies. Tensile, double cantilever beam, bent beam, and fatigue tests are among the most common techniques employed to study HE. This article reviews hydrogen diffusion behavior, mechanisms, and characterization techniques.

## 1. Introduction

Hydrogen embrittlement (HE) corresponds to the abrupt degradation of mechanical properties of materials in the presence of hydrogen. HE failure in metals was first recognized by Johnson in 1875 [1] and has been observed in many metallic materials such as steels, aluminum alloys, titanium alloys, and superalloys [2,3,4,5,6,7,8]. This problem in metals has been of great concern in various industries including chemical, petrochemical, power, and marine industries. Hydrogen embrittlement can lead to catastrophic failure in oil and gas pipelines as a result of the presence of sour gas or as a result of the blending of natural gas with hydrogen. It has been generally established that hydrogen may reduce the macroscopic and microscopic tensile strength [9,10,11,12,13,14], fatigue strength [15,16,17], and fracture toughness [18,19,20,21,22], while its effect on the rate of fatigue crack growth is still debated, depending on the stress ratio level or frequency [23]. Although extensive studies on the hydrogen embrittlement of metals have been carried out, many issues are yet to be understood. The phenomenon of hydrogen damage is a challenging basic research problem. One main reason for the damage caused by hydrogen in metals and alloys is the extremely small size of the hydrogen atom, which makes it move very fast in the metallic lattice. It is therefore not surprising that over the years, a considerable research effort has been directed toward obtaining an understanding of this phenomenon.

Hydrogen-induced failures arise because cracks are able to grow to critical dimensions, with the initial stress intensity level increasing to the point under the requirement that K = K_IC_, where K is stress intensity factor and K_IC_ is the critical stress intensity factor. Such crack extension can occur through a number of processes. Subcritical flaw growth mechanisms involving a cooperative interaction between a stress and the environment, leading to hydrogen embrittlement, and the final failure typically occurs after a period of time, rather than when exposure begins. This damage mechanism affects many important alloy systems, most notably high-strength steels. When atomic hydrogen is introduced into an alloy, the toughness and ductility can be reduced dramatically, and subcritical crack growth can occur. Body-centered cubic and hexagonal close-packed metals are most susceptible to hydrogen embrittlement. Face-centered cubic metals are not generally susceptible to hydrogen embrittlement. Hydrogen has a very high mobility in the BCC lattice of carbon and low-alloy steels [24].

Recently, there has been a renewed interest in the hydrogen embrittlement of metals as a result of the ever-increasing demand from world governments for cleaner energy. Global gas utility companies are exploring ways to blend natural gas with hydrogen as a cleaner energy source. However, the effect of hydrogen on existing infrastructure, including existing and new pipe networks, needs to be assessed prior to injecting hydrogen into the system, and the maximum hydrogen addition for safe operation needs to be determined. This is especially urgent and essential for older distribution pipeline networks, as pipe steels are known to be susceptible to hydrogen embrittlement, which may lead to catastrophic failures. 

In this review, three aspects of the HE behavior of metals are discussed: hydrogen diffusion behavior, hydrogen embrittlement mechanisms, and HE characterization methods.

## 2. Entry of Gaseous and Aqueous Hydrogen into Metals

The exploration in this section is centered around the diffusion process of hydrogen from aqueous and gaseous media into metals. Metal surfaces exhibit a tendency of adsorption. This tendency stems from the fact that while the metal atoms inside the metal are in equilibrium with each other, the metal atoms located on the surface of the metal are not, leading to the manifestation of surface energy on the surface of the metal [25,26]. According to the second law of thermodynamics, the energy of all systems is inherently tilted toward lower values, so that on the surface of metals, hydrogen-containing substances tend to be adsorbed to reduce the overall energy of the system [27].

**Figure 1:**
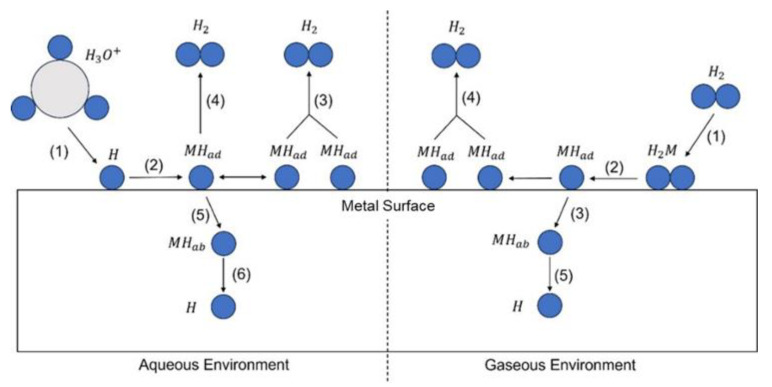
Possible ways of hydrogen transport and interaction in steel.

The primary gateway for hydrogen into metals is through surface adsorption, a process where certain solids selectively concentrate particular substances from a solution (gas or liquid) onto their surfaces [28]. Hydrogen diffusion into metals involves three principal mechanisms. These are physisorption (physical adsorption), chemisorption (chemical adsorption), and hydrogen uptake. Physisorption is typically created by van der Waals forces between hydrogen and the metal surface. This adsorption process is reversible and is primarily influenced by the conditions of the environment, such as pressure and temperature. The second mechanism is chemisorption, which involves the formation of a typically covalent chemical bond between molecules or atoms and is generally irreversible [29,30]. The final mechanism is hydrogen uptake, where hydrogen diffuses into the metal through desorption, leading to its incorporation into the metal lattice. Figure 1 provides an illustration of this concept.

The aqueous hydrogen diffusion process can be primarily depicted by the Volmer–Tafel–Heyrovsky reaction mechanism, which includes several significant stages [31,32,33,34]:

Electrochemical reduction: the initial stage of the reaction involves the reduction of hydronium ions ($H_{3}O^{+}$) by gaining electrons to produce water and atomic hydrogen. The reaction is represented as follows:

(R1) $$H_{3}O^{+}+e^{-}\overset{\;}{\to }H_{2}O+H$$

Volmer reaction (chemisorption): Subsequently, the atomic hydrogen produced interacts with the metal surface, resulting in chemisorption. This process, also known as the Volmer reaction, generally occurs when the overpotential is relatively low due to a limited surface coverage of hydrogen [35].

(R2) $$H+M\overset{\;}{\to }MH_{ad}$$

Tafel reaction: adsorbed hydrogen *(*$MH_{ad}$*)* can recombine and create molecular hydrogen.

(R3) $$2MH_{ad}\overset{\;}{\to }2M+H_{2}$$

Heyrovsky reaction: When overpotential increases due to substantial hydrogen presence on the metal surface, the Heyrovsky reaction prevails [35]. In this scenario, most atomic hydrogen generates gaseous hydrogen and leaves the metal surface. This reaction can be expressed as follows:

(R4) $$MH_{ad}+H_{2}O+e^{-}\overset{\;}{\to }M+H_{2}+OH^{-}$$

Hydrogen absorption: while the reactions expressed in (3) and (4) are taking place, the absorption process is also in progress, where the atomic hydrogen is absorbed into the inner surface of the metal, represented by the following:

(R5) $$MH_{ad}\overset{\;}{\to }MH_{ab}$$

Desorption and dissolution: The final stage involves the desorption of the absorbed atomic hydrogen inside the metal. It is worth noting that this step is less about desorption and more about the dissolution of hydrogen into the metal lattice. This process can induce various microstructural changes and potentially lead to hydrogen embrittlement.

(R6) $$MH_{ab}\overset{\;}{\to }M+H$$

The process of hydrogen diffusion in gaseous media parallels that of aqueous hydrogen diffusion, and it unfolds as follows [30,36,37]:

Physisorption: The initial stage of the process begins when gaseous hydrogen comes into contact with the metal surface, resulting in physisorption. During this phase, no chemical bonds are formed between the hydrogen and metal, making it a weaker form of adsorption. Hence, the adsorption process is reversible.

(R7) $$H_{2}+M\overset{\;}{\to }H_{2}M$$

Chemisorption: following physisorption, the $H_{2}M$ and the metal surface form chemical bonds, marking an irreversible process.

(R8) $$H_{2}M+M\overset{\;}{\to }2MH_{ad}$$

Absorption: subsequent to the chemisorption phase, the adsorbed hydrogen ($MH_{ad}$*)* is absorbed into the subsurface of the metal, resulting in the formation of absorbed hydrogen *(*$MH_{ab}$).

(R9) $$MH_{ad}\overset{\;}{↔}MH_{ab}$$

Recombination: simultaneously, the adsorbed hydrogen can also recombine to generate molecular hydrogen ($H_{2}$).

(R10) $$2MH_{ad}\overset{\;}{\to }2M+H_{2}$$

Desorption and dissolution: the absorbed hydrogen ($MH_{ab}$) undergoes a desorption process, transforming into atomic hydrogen dissolved within the metal, as given in R6.

The above only describes how hydrogen enters the metal in the aqueous and gaseous environments; knowledge about the reaction kinetics is excluded in this paper, and readers who are interested in this knowledge can refer to the work of Popov et al. [34].

## 3. Mechanisms of Hydrogen Diffusion

Due to their very small atomic size, hydrogen atoms have a greater tendency to diffuse or dissolve into steel compared to other atoms. Two primary mechanisms that govern hydrogen diffusion in metals are interstitial diffusion and quantum mechanical tunnel diffusion [38]. Given the high diffusivity of hydrogen in steel and the significant alteration it can cause in the metal’s mechanical properties, it is critical to understand the mechanisms of hydrogen diffusion in metals in detail. Firstly, the process of interstitial diffusion of hydrogen is examined.

**Figure 2:**
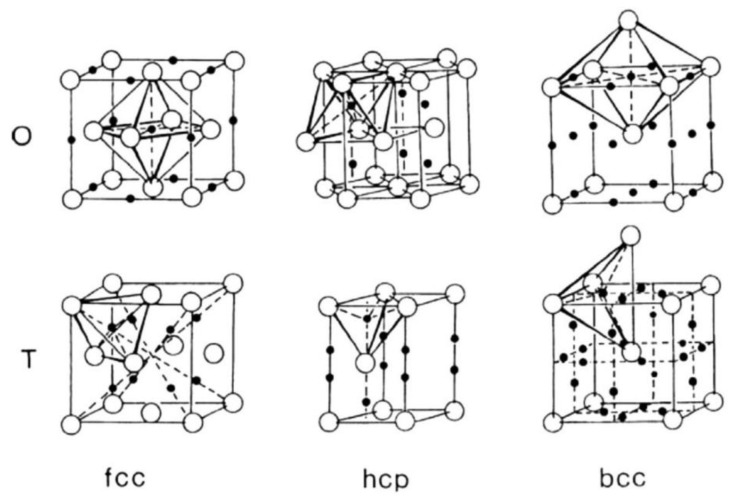
Interstitial sites (octahedral (O) sites and tetrahedral (T) sites) in fcc, hcp, and bcc lattices [43].

For the metals considered in this section, it is vital to emphasize that they are devoid of defects, meaning hydrogen atoms are not held in hydrogen traps. The effect of hydrogen traps on hydrogen diffusion is elaborated upon in Section 5.1 of this article. As shown in Figure 2, in the three main crystal structures—face-centered cubic (fcc), hexagonal close-packed (hcp), and body-centered cubic (bcc)—there are two types of interstitial sites, octahedral (O) and tetrahedral (T), that can house hydrogen. The absorption capacity of O sites and T sites varies and primarily hinges on the size of their interstitial gaps. An approximation based on the diameter of hydrogen atoms and the size of these gaps suggests that O sites are primarily populated in fcc and hcp structures, while T sites are more frequent in bcc structures. Elevated mobilities are particularly prevalent in body-centered cubic (bcc) metals due to the presence of adjacent interstitial sites that are in close proximity [38]. As hydrogen atoms dissolve in the metal, these sites progressively fill, displacing metal atoms and leading to elastic distortion and changes in crystal entropy. This effect translates to an alteration in enthalpy. The system’s energy$\;\left(\Delta G\right)$ is at its minimum when hydrogen atoms reside in the interstitial sites and peaks when the hydrogen atoms are positioned between metal atoms. This peak value can be represented in terms of this enthalpy change. Consequently, the diffusion of hydrogen atoms occurs only when they are thermally activated and the energy that they possess exceeds ΔG.

Nonetheless, quantum mechanics suggests that diffusion can still occur when the energy of a hydrogen atom is lower than this energy barrier, a phenomenon known as quantum mechanical tunnel transport [39]. Due to its diminutive size, treating the hydrogen atom solely as a classical particle is inaccurate [40]. This topic has been the subject of extensive research, with scholars finding that this quantum effect becomes less significant at higher temperatures, with normal diffusion mechanisms taking precedence. At these temperatures, hydrogen atoms must surpass the energy barriers to diffuse. However, the impact of quantum mechanical tunnel transport becomes more pronounced at lower temperatures. Some researchers have postulated this temperature threshold to be around 250 K [41]. This suggests that quantum mechanical tunnel transport is not thermally activated, leading to the possibility that diffusion models based on the Arrhenius equation might be incorrect [42].

## 4. Characterization Techniques for the Measurement of Hydrogen Diffusion

### 4.1. Hydrogen Microprint Technique

**Figure 3:**
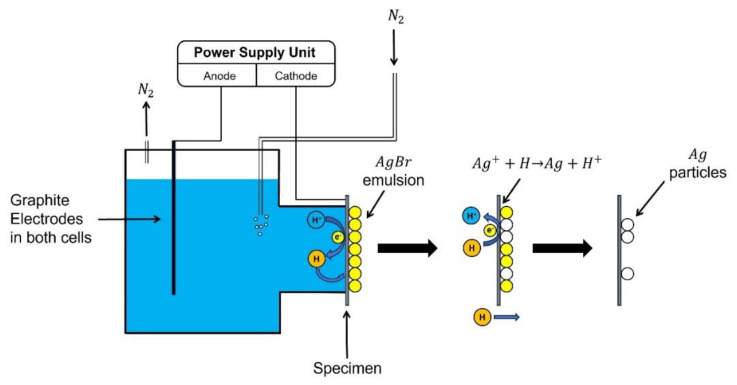
Schematic of the HMT test setup [45].

The hydrogen microprint technique (HMT), a method commonly used for visualizing hydrogen diffusion in metals, was developed by Ovejero-García [44]. The foundational principle of this technique involves generating silver (Ag) microparticles via the reduction of silver bromide (AgBr) on the surface of a coated specimen. These silver microparticles can then be observed using scanning electron microscopy (SEM), providing insights into the behavior of hydrogen diffusion. The schematic and underlying principle of the experimental apparatus are illustrated in Figure 3.

The implementation of the hydrogen microprint technique (HMT) initiates with specific preparatory steps: Initially, one surface of the specimen is ground to achieve a 600-grit finish, while the side exposed to air is polished to a 1 μm scale using diamond paste. If examination of the steel’s microstructure is intended, the polished side of the specimen requires etching for a duration of 30 s using a 2% nital solution. Following this, a layer of silver bromide (AgBr) nuclear emulsion is applied to the polished surfaces, which is then left to dry for a period of 20 min or longer. This emulsion comprises 5 g of AgBr powder and a 10 mL solution of 1.4 M sodium nitrite ($NaNO_{2}$). The employment of sodium nitrite serves a specific purpose, which is to mitigate corrosion of the etched side during the experiment.

**Figure 4:**
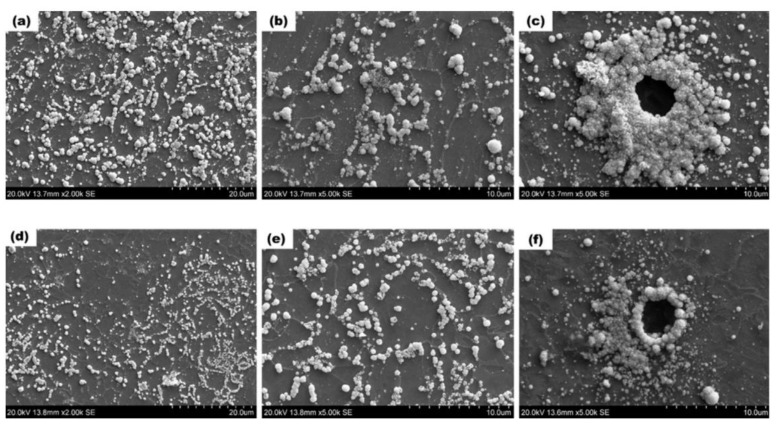
SEM images of the HMT test [45].

The specimen is positioned in the apparatus as shown in Figure 3, with the side devoid of AgBr exposed to the electrolyte for charging. This initiates a process analogous to the reaction occurring on the charging side during permeation tests, where hydrogen atoms diffuse to the surface of the specimen and permeate through it. Hydrogen atoms diffusing from the opposite side then interact with silver ions ($Ag^{+}$) according to the reaction $Ag^{+}+H\underset{\;}{\to }Ag+H^{+}$. This results in the reduction of Ag to visible silver particles, as depicted in Figure 3. Following a charging duration of 80 min or longer, the specimen is retrieved and immersed for 1 min in a photographic fixing solution composed of 0.6 M sodium thiosulfate ($Na_{2}S_{2}O_{3}$) and 1.4 M sodium nitrite ($NaNO_{2}$). This crucial step serves to eliminate any unreacted silver bromide (AgBr) crystals. After a final rinse with deionized water and dehydration using a dryer, the resultant silver particles become visible. These particles, which appear as white spheres on the microstructure, are then ready for observation. Consequently, the diffusion of hydrogen to the steel surface can be effectively visualized using scanning electron microscopy (SEM) [44,45,46,47]. Jack et al.’s [45] SEM observations are depicted in Figure 4. Their experiments, conducted on two samples simultaneously, reveal distinct white spheres in the microstructure, corresponding to the reduced Ag particles. These particles signify hydrogen permeation through grain boundaries and phase interfaces. Figure 4c,f potentially exhibit matrix distortion and high local misorientation, commonly observed around inclusions.

### 4.2. Hydrogen Permeation Tests

#### 4.2.1. Electrochemical Permeation

**Figure 5:**
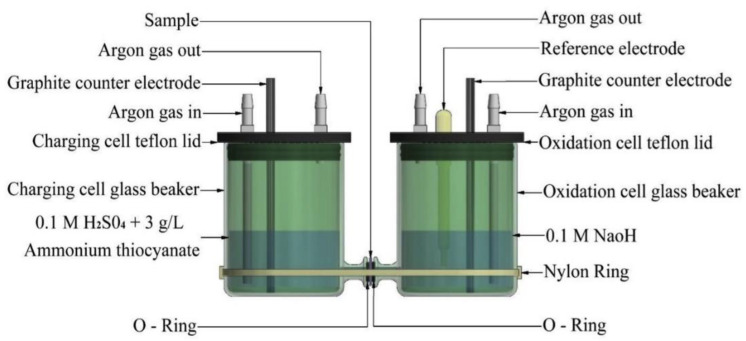
Schematic of electrochemical permeation test [46].

While the hydrogen microprint technique (HMT) allows for the visualization of hydrogen distribution within a steel microstructure, it falls short in quantifying the hydrogen permeation process. To address this shortcoming, many researchers have adopted the methodology pioneered by Devanathan and Stachurski [48]. A representation of their experimental setup is illustrated in Figure 5. Below is a step-by-step breakdown of the procedure.

Initially, the test specimen is polished and cleaned. Subsequently, materials such as palladium or other suitable alternatives (like nickel) are used to coat the side of the specimen exposed to the oxidation cell, thereby preventing oxidation and reaching a steady permeation state in a shorter time, and improving the stability of current measurement [49,50]. Once the specimen is prepared, it is clamped between the charging and oxidation cells.

**Figure 6:**
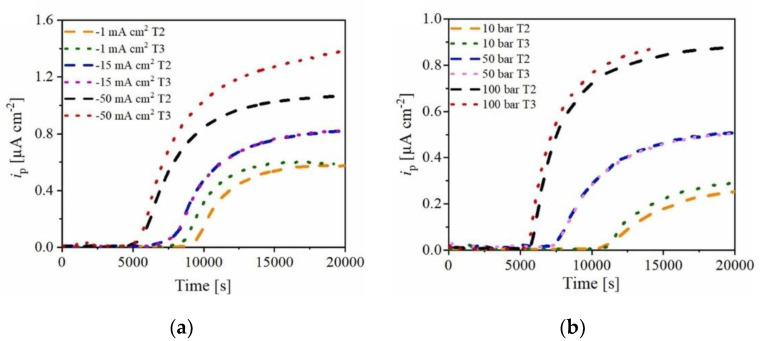
(**a**) Electrochemical hydrogen permeation curves of different charging currents; (**b**) gaseous hydrogen permeation curves of different charging hydrogen pressure [55].

For the charging cell, the electrolyte can be either acidic or basic. Widely used electrolytes include $0.1\;M\;H_{2}SO_{4}$ or 0.1 M NaOH, but the pH of the electrolyte can influence the experimental results [51]. Additionally, a recombination poison is introduced to the charging side to prevent hydrogen atoms in the charging cell from recombining into $H_{2}$. Ammonium thiocyanate ($NH_{4}SCN$) is commonly used, with its concentration affecting the permeation current [52]. Traditionally, a concentration of 3 g/L is used [46,53]. Lu et al. have also incorporated 0.2 g/L thiourea ($CH_{4}N_{2}S$) [32]. Apart from the aforementioned conditions, there have been instances, such as the work of Fallahmohammadi et al., where a solution containing $0.2\;M\;CH_{3}COOH$ and $0.4\;M\;CH_{3}COONa$ (pH = 4.2) was employed [54]. The power supply unit (PSU) has its anode connected to a graphite electrode or any other inert electrode such as platinum (Pt) and its cathode connected to the specimen. As for charging conditions, both galvanostatic and potentiostatic power supply units (PSUs) can be utilized. The chosen currents and voltages will dictate the quantity of hydrogen atoms generated. Evidently, a higher current or potential result in an increased production of hydrogen, which leads to a higher steady-state current, as illustrated in Figure 6. Additionally, using potentiostatic charging allows for the analysis of the equivalence between gaseous hydrogen permeation and electrochemical hydrogen permeation. Further discussions on this topic can be found in Section 4.2.3 of this article.

In the oxidation cell, studies typically use 0.1 M NaOH as the electrolyte. This cell comprises a three-electrode system with an electrochemical working station. The test specimen acts as the working electrode, a saturated calomel electrode (SCE) serves as the reference electrode, and an inert electrode functions as the counter electrode. The primary role of the electrochemical working station is to monitor the oxidation current.

After assembly, the electrolyte is first introduced into the oxidation cell, and an inert gas is passed through. The charging cell is kept idle until the measured current decreases below $0.1\;\mu A/{\mathrm{cm}}^{2}$, which signifies the background current [50]. This current is later subtracted from the oxidation current during the analysis to obtain the actual permeation current. This step aims to remove any residual hydrogen present in the metal from prior processing. Once the process is finalized, the electrolyte mixed with recombination poison is added into the charging cell and the power supply is activated. Depending on the pH of the electrolyte, acidic or alkaline, $H_{2}O$ or $H^{+}$ is reduced. This process involves these molecules gaining electrons to produce hydrogen atoms. When these atoms permeate through the specimen and reach the surface of the oxidation side, they are oxidized, generating an oxidation current measured by the electrochemical working station. The experiment is typically conducted at room temperature.

#### 4.2.2. Gaseous Permeation

While electrochemical hydrogen charging predominates due to its relative simplicity and safety, gaseous hydrogen charging presents its own set of unique benefits and challenges [2,49,55]. The primary challenges associated with gas-phase charging include the following:

1. The need for a dedicated gas line system, with specialized valves and pressure gauges.
2. An intricate, tightly sealed arrangement to prevent any potential hydrogen leakage.
3. All components must be resilient against hydrogen embrittlement and able to withstand test pressures.
4. Presence of hydrogen in the charging cell prior to the completion of pressurization, can lead to inaccurate measurements.
5. For experiments across a spectrum of temperatures, the test apparatus must be robust enough to withstand such temperature fluctuations.

**Figure 7:**
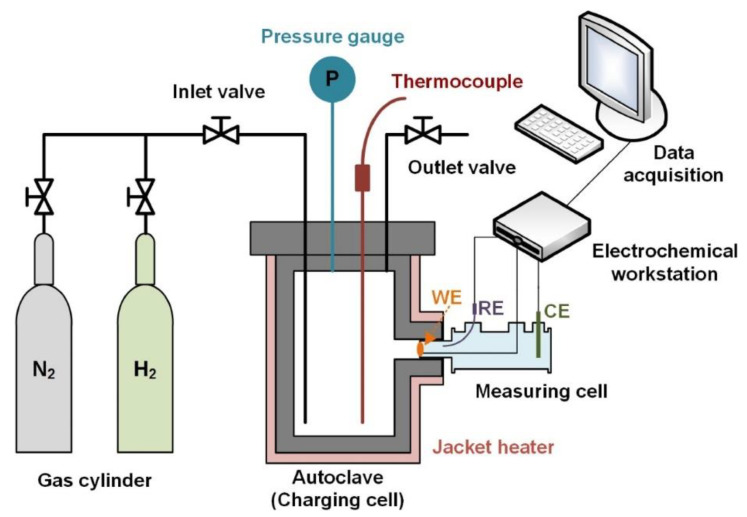
Schematic of gaseous hydrogen charging chamber [57].

One of the main advantages of the gaseous hydrogen permeation test is its capability to mimic real-world environments. The experimental outcomes are often more representative of practical applications. Figure 7 showcases a typical setup for this method, which bears significant resemblance to the electrochemical permeation test.

In this experiment, an inert gas, such as nitrogen ($N_{2}$), is used to purge the test chamber prior to testing. This is followed by a background current-obtaining procedure, after which hydrogen gas is introduced to generate the permeation current. The pressure at which hydrogen is introduced directly impacts the quantity of hydrogen atoms formed on the charging side. Consequently, a higher pressure leads to a larger resultant permeation current. As depicted in Figure 6, this procedure is similar to electrochemical hydrogen charging. The hydrogen gas introduced can be varied in terms of its concentration or pressure. It can also be mixed with other gases to yield different blend concentrations.

For instance, Zhang et al. performed a permeation test using different concentrations of hydrogen and nitrogen. They also performed fracture toughness and fatigue tests using a similar blend ratio. It was found that 3% hydrogen in the blended gas can cause 67.7% reduction in fatigue life [56]. Exploring an alternative approach, Zhao et al. created a simulated coal gas by combining nitrogen, CO_2_, and hydrogen. Using this simulated gas, they conducted tests to measure hydrogen permeation and explored slow strain rate tension tests (SSRT). Their investigation identified the coarse-grained heat-affected zone (CGHAZ) of X80 as the region with the highest hydrogen diffusion velocity. This increased velocity led to a rapid accumulation of hydrogen near the crack’s front, enhancing the material’s susceptibility to hydrogen embrittlement [49].

In conclusion, although both of these procedures are different, they also share core similarities, with both ultimately producing a permeation current. Depending on the test procedure used, it is possible to gain profound insights into the hydrogen permeation behavior.

#### 4.2.3. Permeation Test Results Analysis

Whether in water or gaseous environments, hydrogen diffuses due to the concentration gradient. During this process, hydrogen atoms migrate from areas of high concentration to low concentration. Understanding the concentration gradient is essential for the study of hydrogen diffusion dynamics. The principles of this model can be formulated via Fick’s First and Second Laws. Fick’s First Law describes the diffusion flux, i.e., the relationship between the rate of substance flow and the concentration gradient, which is expressed by Equation (1):

(1) $$\begin{array}{c} J=-D\frac{\partial c}{\partial x}\; \end{array}$$

where J represents the diffusion flux, D represents the diffusion coefficient, and ∂c/∂x is the concentration gradient. The negative sign in Equation (1) indicates that the diffusion flux always flows from regions of higher concentration to lower concentration, in opposition to the direction of the increasing concentration gradient.

Fick’s Second Law, on the other hand, accounts for the changes in concentration over time and across the diffusion distance. It is expressed by Equation (2), where ∂c/∂t represents the change in concentration over time, D is the diffusion coefficient, and $\partial^{2}c/\partial x^{2}$ is the second derivative of the concentration with respect to the distance, capturing the spatial variation in the concentration gradient.

(2) $$\begin{array}{c} \frac{\partial c}{\partial t}=D\frac{\partial^{2}c}{\partial x^{2}}\; \end{array}$$

**Figure 8:**
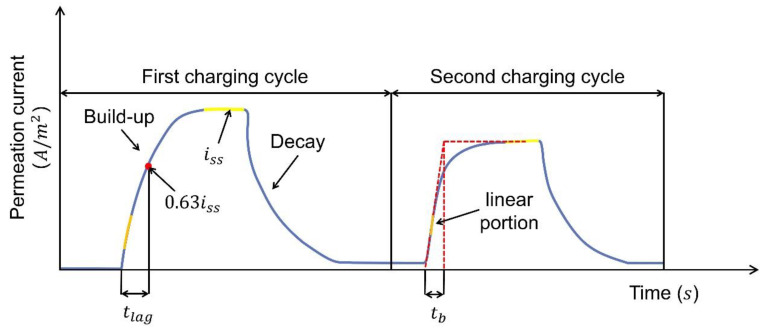
Typical hydrogen permeation curve during first and second charging cycles [46].

Figure 8 shows a schematic of permeation test results after the first and second cycle. Hydrogen concentration on both sides of the specimen is zero prior to the experiment. After achieving a steady state (represented by the plateau), the hydrogen concentration on the entry side (previously referred to as the charging side) is denoted as $c_{0R}$, while on the exit side (previously referred to as the oxidation side), it is zero. Equations (3)–(8) can be derived by applying Fick’s First and Second Laws [49,54,58]:

Time lag method for $D_{eff}$ calculation:

(3) $$\begin{array}{c} D_{eff}=\frac{L^{2}}{6t_{lag}}\; \end{array}$$

Breakthrough time method for $D_{eff}$ calculation:

(4) $$\begin{array}{c} D_{eff}=\frac{L^{2}}{15.3t_{b}}\; \end{array}$$

Fourier method for $D_{eff}$ calculation:

(5) $$\begin{array}{c} \frac{i_{t}}{i_{\mathrm{ss}}}=1-2\exp\left(-\frac{\pi^{2}D_{eff}t}{L^{2}}\right)\; \end{array}$$

Laplace method for $D_{eff}$ calculation:

(6) $$\begin{array}{c} \frac{i_{t}}{i_{\mathrm{ss}}}=\frac{2}{\sqrt{\pi }}\frac{L}{\sqrt{D_{eff}t}}\exp\left(-\frac{L^{2}}{4D_{eff}t}\right)\; \end{array}$$

Hydrogen permeation flux in the steady state:

(7) $$\begin{array}{c} J_{SS}=\frac{i_{SS}/A}{F}=\frac{D_{eff}C_{0R}}{L}\; \end{array}$$

Subsurface hydrogen concentration:

(8) $$\begin{array}{c} c_{0R}=\frac{J_{SS}L}{D_{eff}}\; \end{array}$$

In Equations (3)–(8), $i_{t}\;\left(\mu A\right)$ is the transient current and $i_{\mathrm{ss}}\;\left(\mu A\right)$ denotes the steady-state current. $t_{lag}\;\left(s\right)$ is the time elapsed from the beginning of the experiment until the ratio of transient-to-steady-state current $\left(i_{t}/i_{\mathrm{ss}}\right)$ reaches 0.63. $t_{b}\;\left(s\right)$ is the time calculated by extrapolating the linear portion of the rising hydrogen permeation current transient. All of these parameters are marked in Figure 8. $D_{eff}\;\left(m^{2}\cdot s^{-1}\right)$ refers to the effective diffusion coefficient. $J_{SS}\;\left(\mathrm{mol}\cdot m^{-2}s^{-1}\right)$ corresponds to the permeation flux in the steady state, which reflects the rate of hydrogen permeation through the sample under steady conditions. $C_{0R}\;\left(\mathrm{mol}\cdot m^{-3}\right)$ is the summation of the subsurface concentration of hydrogen in interstitial lattice sites and reversible trap sites on the charging side of the sample [59].

Apart from calculating $D_{eff}$ using Equations (3) and (4), this coefficient value can alternatively be estimated using Equation (5) or Equation (6) [58]. This involves constructing a linear graph and ascertaining its slope. For instance, in the case of Equation (5), the equation could be reformulated as Equation (9):

(9) $$\begin{array}{c} \ln\left(1-\frac{i_{t}}{i_{\mathrm{ss}}}\right)=-\frac{\pi^{2}D_{eff}t}{L^{2}}+\ln2 \end{array}$$

In that case, a plot of $\ln\left(1-\frac{i_{t}}{i_{\mathrm{ss}}}\right)$ against t can be established. The effective diffusion coefficient $D_{eff}$ can subsequently be determined by calculating the slope, $-\frac{\pi^{2}D_{eff}}{L}$. In some studies, researchers also turn to fitting the build-up and decay curves described in Equations (10) and (11) to evaluate $D_{eff}$ [54,55,60]. $i_{0}\;\left(\mu A\right)$ is the background current. The build-up and decay curves are noted in Figure 8.

Build-up curve:

(10) $$\begin{array}{c} \frac{i_{t}-i_{0}}{i_{ss}-i_{0}}=\frac{2L}{\sqrt{\pi Dt}}\sum_{n=0}^{\infty }exp\left(-\frac{{\left(2n+1\right)}^{2}L^{2}}{4D_{eff}t}\right)\; \end{array}$$

Decay curve:

(11) $$\begin{array}{c} \frac{i_{t}-i_{ss}}{i_{0}-i_{ss}}=1-\frac{2L}{\sqrt{\pi Dt}}\sum_{n=0}^{\infty }exp\left(-\frac{{\left(2n+1\right)}^{2}L^{2}}{4D_{eff}t}\right)\; \end{array}$$

The calculated values of the $D_{eff}$, derived from these distinct methods, do not coincide, a discrepancy that could potentially be attributed to the phenomenon of short-circuit diffusion that occurs during the hydrogen transport process [61]. To better calculate the $D_{eff}$, the built-up curve in the first charging cycle is typically not preferred in these calculations. This preference stems from the understanding that during the build-up phase of the initial hydrogen charging, hydrogen is ensnared by both irreversible and reversible traps. For the subsequent curves, it is primarily the reversible traps that trap or release the hydrogen. Given that irreversible hydrogen traps are seldom considered in some applications, the $D_{eff}$ ascertained from the first decay curve or second permeation curve offers greater relevance and accuracy for research [49,57,59,62]. Thus, employing the decay curve or second charging curve as the reference often leads to a more exact determination of the $D_{eff}$.

A second charging curve can not only determine a better $D_{eff}$, but also can give an estimation of reversible and irreversible hydrogen trap density. This can provide a differential impact of reversible and irreversible hydrogen traps on hydrogen permeation or HE susceptibility [45,53]. Detailed mechanisms of hydrogen trapping is covered in Section 5.1 and Section 5.2. The estimation of these traps can be calculated from Equation (12), where $N_{T}$ is the total density of hydrogen trap, $N_{A}$ is Avogadro’s number ($6.022\times 10^{23\;}\;{\mathrm{mol}}^{-1}$), and $D_{l}$ is the lattice diffusion coefficient of hydrogen [63].

(12) $$\begin{array}{c} N_{T}=\frac{N_{A}C_{0R}}{3}\left(\frac{D_{l}}{D_{eff}}-1\right) \end{array}$$

If the binding energy ($E_{b}$) is considered, $N_{T}$ can also be calculated by Equation (13), where $N_{L}$ is the density of the interstitial sites in the steel, R is the gas constant ($8.314\;J\cdot K^{-1}\cdot {\mathrm{mol}}^{-1}$), and T is the temperature [64,65,66].

(13) $$\begin{array}{c} N_{T}=N_{L}\times \left(\frac{D_{l}}{D_{eff}}-1\right)\times e^{-\frac{E_{b}}{RT}} \end{array}$$

From prior discussions, it is evident that the $D_{eff}$ derived from the permeation curve of the first hydrogen charging cycle, when applied to either Equation (12) or Equation (13), offers a measure of the total hydrogen trap density ($N_{T}$), given all traps are actively involved hydrogen trapping. On the other hand, using the $D_{eff}$ from the permeation curve of the second hydrogen charging in Equation (12) or Equation (13) provides the density of just the reversible hydrogen traps ($N_{r}$), as only reversible traps are involved in hydrogen trapping. The density of irreversible traps ($N_{ir}$) can be deduced by computing the difference, $N_{T}-N_{r}$ [63].

Apart from Equation (8), the concentration of hydrogen dissolved in a metal can also be quantified using Sieverts’ law:

(14) $$\begin{array}{c} c_{H}=S\times \sqrt{p_{H_{2}}}\; \end{array}$$

(15) $$\begin{array}{c} S=Ae^{-\frac{\Delta H}{RT}}\; \end{array}$$

In Equations (14) and (15), $p_{H_{2}}$ represents the partial pressure of gaseous hydrogen. The term S remains steady when the temperature, denoted by T, is constant. The symbol ΔH represents the dissolution enthalpy and A is the constant. These two parameters can be determined from the graph slope of $\ln\left(c_{H}\right)$ against 1/T. However, when the hydrogen pressure exceeds a threshold of around 200 atm (approx. 20 MPa), the hydrogen concentration is determined by Equations (16) and (17). $f_{H_{2}}$ is the fugacity, which is defined as the pressure of an ideal gas with the same chemical potential as the real gas. The calculation of fugacity aligns with Equation (17), recognized as the Able–Noble relationship. In this equation, b stands as a constant with a value set at $1.584\times 10^{-5}\;m^{3}{\mathrm{mol}}^{-1}$ [67,68,69]:

(16) $$\begin{array}{c} c_{H}=S\cdot \sqrt{f_{H_{2}}}\; \end{array}$$

(17) $$\begin{array}{c} f_{H_{2}}=p_{H_{2}}\cdot \exp\left(\frac{p_{H_{2}}b}{RT}\right) \end{array}$$

A group of researchers established a relationship between gaseous charging and electrochemical charging, and they believe that the two charging conditions can be treated as analogous processes if two conditions can produce the same fugacity of hydrogen in metal [32,35,55,70,71,72]. For electrochemical charging, hydrogen fugacity can be calculated according to Equations (18)–(20). In these equations, η is the overpotential of the hydrogen evolution reaction; A and ζ are constants which are determined by the mechanism of hydrogen evolution reaction. $E_{H}^{0}$ stands for the equilibrium potential at the surface of the steel in the hydrogen evolution reaction’s charging solution under a fugacity of 1 atm. Meanwhile, $E_{C}$ represents the potential that has been applied to the specimen and counter electrode. It should be noted that $f_{H_{2}}$ in Equation (20) is 1 atm, so $\log\left(f_{H_{2}}\right)$ equals 0 [35,68].

(18) $$\begin{array}{c} f_{H_{2}}=A\cdot \exp\left(\frac{-\eta F}{\zeta RT}\right)\; \end{array}$$

(19) $$\begin{array}{c} \eta =E_{C}-E_{H}^{0}\; \end{array}$$

(20) $$\begin{array}{c} E_{H}^{0}=-0.0591\times pH-0.0295log\left(f_{H_{2}}\right)\; \end{array}$$

## 5. Factors Affecting Hydrogen Diffusion into Metals

### 5.1. Hydrogen Trapping

#### 5.1.1. Hydrogen Trapping Mechanism

**Figure 9:**
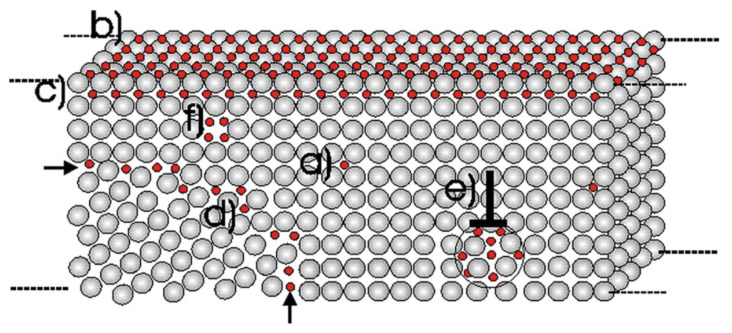
Schematic of hydrogen trapped in steel: (**a**) interstitial sites, (**b**) surface, (**c**) subsurface, (**d**) boundary sites, (**e**) edge dislocations (position indicated by ⊥), and (**f**) vacancies [38].

Hydrogen traps fundamentally refer to a variety of crystal defects in metals capable of binding with hydrogen. This binding prolongs the interaction between hydrogen and the metal, affecting the metal’s hydrogen permeability and consequently its vulnerability to hydrogen embrittlement. The range of crystal defects in metals is broad, starting with point defects which include vacancies and diverse solute atoms. Beyond point defects, there are line defects, embodied by entities such as dislocations. Metals also feature plane defects, predominantly grain boundaries. Finally, volumetric defects are observed, comprising inclusions, precipitation phases, voids, and different crystallographic phases. It is important to note that hydrogen atoms trapped in vacancies can lead to the creation of voids, and subsequently, molecular hydrogen can be formed [73]. This mechanism is illustrated in Figure 9.

To better describe the behavior of hydrogen trapping, Lee et al. provided the reaction equation for the process of trapped hydrogen detaching from the trap and entering the lattice interstitial positions [74]:

(R11) Htrap↔  ☐trap+Hlattice 

In this equation, ${☐}_{trap}$ represents the hydrogen trap, Htrap denotes the hydrogen in the trap with its concentration represented by $C_{T}$, and $H_{lattice}$ indicates the hydrogen at lattice interstitial sites, with its concentration represented by $C_{L}$. Lu et al. [32] formulated equations to describe this process, as shown in Equations (21)–(26):

(21) $$\begin{array}{c} \theta_{L}=\frac{C_{L}}{N_{L}}\; \end{array}$$

(22) $$\begin{array}{c} \theta_{trap}=\frac{C_{T}}{N_{T}}\; \end{array}$$

(23) $$\begin{array}{c} N_{L}=\frac{N_{A}\beta \rho }{A}\; \end{array}$$

(24) $$\begin{array}{c} \frac{\theta_{trap}}{1-\theta_{trap}}=\theta_{L}\cdot exp\left(-\frac{E_{b}}{RT}\right) \end{array}$$

(25) $$\begin{array}{c} \frac{C_{T}}{C_{L}}=\frac{N_{T}}{N_{L}}\cdot exp\left(-\frac{E_{b}}{RT}\right)\; \end{array}$$

(26) $$\begin{array}{c} \frac{C_{L}}{N_{L}}\approx exp\left(-\frac{E_{L}}{RT}\right)\; \end{array}$$

In the provided equations, $\theta_{L}$ denotes the occupancy of lattice sites and $N_{L}$ represents the number of lattice sites per unit volume. Other parameters include $N_{A}$, which is Avogadro’s number defined as $6.02\times 10^{23}\;{\mathrm{mol}}^{-1}$; β, the number of interstitial sites per atom; ρ, the alloy’s density; and A, the atomic weight of atoms in the alloy. Furthermore, $E_{b}$ stands for the binding energy of the hydrogen trap, and $E_{L}$ is the solubility energy of interstitial hydrogen. It is notable that if $\theta_{trap}$ is much less than 1, then Equation (24) simplifies to Equation (25). The concentration of lattice hydrogen is shown to follow a straightforward statistical distribution, as demonstrated in Equation (26). $E_{b}$ and $E_{L}$ can be determined by plotting $ln\left(\frac{C_{T}}{C_{L}}\right)$ and $ln\left(\frac{C_{L}}{N_{L}}\right)$ against the reciprocal of temperature, $\frac{1}{T}$ [8]. In Equation (25), it is not difficult to find that the lower the $E_{b}$ or the higher the T, the lower the $C_{T}$ [75].

#### 5.1.2. Classification of Hydrogen Traps

Hydrogen traps can be classified into reversible and irreversible categories, a classification based on the magnitude of the binding energy, $E_{b}$. Some researchers suggest that this categorization is driven more by practical implications, not solely by whether the binding energy surpasses or is less than a certain threshold [59]. For example, according to Equation (25), there is a decrease in the concentration of hydrogen contained within traps as the temperature escalates. This suggests that while certain traps might exhibit reversibility at elevated temperatures, they act irreversibly at typical operational temperatures. Consequently, such traps should be categorized under irreversible hydrogen traps. Indeed, this phenomenon underscores one of the pivotal factors highlighting the temperature’s role in influencing the hydrogen permeation process [76]. On the other hand, some researchers advocate that traps with a binding energy ($E_{b}$) greater than 55 kJ/mol should be defined as irreversible traps [77].

At present, many researchers posit that reversible hydrogen traps play a more significant role in elevating the susceptibility to hydrogen-induced cracking (HIC) [45,78]. In contrast, irreversible traps, due to their reduced accumulation of free hydrogen at the sites of crack initiation, are perceived to possess a lower likelihood of instigating HIC [59,79].

#### 5.1.3. Effects of Hydrogen Traps on Hydrogen Diffusion Behavior

##### Solute-Atom Hydrogen Traps

Different types of hydrogen traps manifest distinct diffusion behaviors, as extensively explored by previous researchers. Primarily, in the context of solute-atom hydrogen traps, alloys exhibit a lower rate of diffusion compared to pure metals due to the presence of a larger number of hydrogen traps. Fu and colleagues have demonstrated that the influence of different atomic dopants on hydrogen diffusion varies. Specifically, the addition of elements such as C, Si, and Mo can enhance the solubility of hydrogen in Fe. In contrast, the incorporation of Mn and Cr has the opposite effect; these elements decrease the solubility of hydrogen. Therefore, they propose that by adjusting the proportions of metal elements, the solubility of hydrogen in metals can be effectively reduced, which can further decrease the susceptibility of the metal to hydrogen embrittlement [80]. Similarly, Beck et al. discovered that a significant amount of hydrogen is trapped in nickel alloys compared to pure iron [81]. Fukuda and colleagues found that in martensite, the quantity of trapped hydrogen elevates with increasing carbon content [82].

Additionally, Pressouyre et al. established that the hydrogen diffusivity in ferrite decreases with an increase in nickel, chromium, and titanium content [83]. Conversely, Zaw et al. argued that the role of titanium in hydrogen trapping is not strictly linear. Their research into the impact of titanium content in vanadium–titanium alloys on hydrogen trapping revealed a complex relationship. With the addition of 0.5% titanium, the amount of trapped hydrogen reduced, whereas with the addition of 1% and 5% titanium, the trapped hydrogen amount increased. However, an addition of 10% titanium predominantly resulted in a decrease in the amount of captured hydrogen [84].

From these studies, it becomes clear that the influence of solute atoms on hydrogen diffusion is not as straightforward as merely reducing the diffusion rate. Rather, it is a multifaceted process significantly impacted by the type and concentration of the solute atoms. Therefore, these variables should be comprehensively considered when conducting research in this area.

##### Grain Size and Grain Boundaries

The influence of grain size and grain boundaries on hydrogen diffusion has also been thoroughly explored. As the grain size decreases, the density of grain boundaries increases. When hydrogen diffuses through a metal, these grain boundaries can often expedite the diffusion process. This is primarily because hydrogen tends to travel along these grain boundaries, a phenomenon referred to as short-circuit diffusion [85]. Even though grain boundaries are often considered hydrogen traps, they can sometimes facilitate faster diffusion, as reported by Oudriss et al. in their study of hydrogen short-circuit diffusion in polycrystalline nickel [86].

However, an increase in grain boundaries has also been observed to raise the density of nodal points, which can subsequently slow down hydrogen diffusion. For instance, Ichimura et al. developed a model of hydrogen diffusivity in aluminum calculated based on different grain sizes. Their findings indicated that for smaller grains, the diffusivity decreases because the grain boundary nodes participate more extensively in trapping at this point. When the grains are larger, hydrogen tends to diffuse along the grain boundaries, leading to an increase in diffusivity [87].

Apart from this, intracrystalline or lattice diffusion also plays a crucial role in the overall diffusion behavior. Therefore, all of these factors—grain size, grain boundary characteristics, and intracrystalline diffusion—should be considered when analyzing hydrogen diffusion in metals.

##### Dislocations

Due to its low binding energy, dislocations can be considered as weak hydrogen traps [88]. Dislocations in metals are primarily generated due to external stresses or cold working, as well as uneven rates during the crystal cooling process [89].

In terms of dislocations produced by external stresses or cold working, when dislocations serve as hydrogen traps, the hydrogen diffusion rate generally decreases. Yunjian and colleagues applied cold work to metals through the method of laser peening (LP) and studied the subsequent permeation rate. It was observed that metals treated with LP exhibited more tortuous grain boundaries and increased dislocations, and the amount of hydrogen permeating into the metal diminished [90]. However, as highlighted by Martin et al., hydrogen demonstrates a pronounced responsiveness to the elastic stress field surrounding dislocations [33]. When external force is exerted, it results in the creation of dislocations in the crystal, prompting hydrogen to migrate along these dislocations—a phenomenon termed as hydrogen–dislocation drag [91,92]. In such scenarios, the interaction between dislocations and hydrogen in metals is multifaceted. Hydrogen capture by dislocations might curtail the diffusion rate, while hydrogen migration with the movement of dislocations could potentially accelerate it. These two mechanisms coexist and compete internally within the metal.

Another significant source of dislocations is the segregation induced by metal cooling. There are primarily two reasons leading to this type of segregation during metal cooling: the differential cooling rates between inclusions and the metal matrix, and the disparate cooling rates at the metal surface compared to its centerline. For example, in casting processes, the exterior of the metal solidifies more rapidly, resulting in a finer grain structure and elevated dislocation density. As a result, the hydrogen diffusion efficacy at the metal’s surface tends to be inferior to that at the centerline [93].

### 5.2. Effect of Microstructure

The intricate microstructures within steel play a pivotal role in dictating its hydrogen permeation characteristics. By subjecting steel to diverse heat treatments, a variety of microstructures emerge. Park et al. [94] treated API X65 grade pipeline steel thermally, obtaining samples spanning various microstructures. Through meticulous hydrogen permeation tests, they gauged the diffusivity of these uniquely structured samples, unveiling an escalating hydrogen permeation rate sequence: acicular ferrite < bainite < degenerated pearlite [94]. This observation aligns with the findings of Thomas et al., who concluded that hydrogen diffusion in X70 pipes primarily occurs through grains, grain boundaries, triple junctions, and cementite. Furthermore, they posited that traversing through cementite in degenerated pearlite represents the least complex diffusion pathway for hydrogen [46]. Thomas’ result is also consistent with the findings of Haq et al. [65].

Another area warranting keen interest is the interplay of phase interfaces amid distinct microstructures. Turnbull et al. pinpointed the austenite/martensite phase juncture as a potent hydrogen-trapping locus [95]. Venturing further, Rudomilova et al. worked with samples enriched with ferrite, martensite, and a trace of residual austenite. Their observations underscored that specimens characterized by a relatively coarse ferrite microstructure exhibited peak diffusivity, potentially influenced by their pronounced grain size and sparser phase interfaces [62]. Offering a comprehensive assessment, Zhou et al. [66] examined the hydrogen permeation dynamics across an array of microstructures: single-phase, dual-phase, and intricate multi-phase configurations. Their study illuminated that phase boundaries such as martensite/ferrite, martensite/austenite, and ferrite/austenite acted as reversible hydrogen traps, impeding hydrogen’s spread. Additionally, they proposed that while filmy retained austenite behaves as a reversible hydrogen trap, its blocky counterpart operates as an irreversible trap. Elevating the austenite phase fraction can potentiate hydrogen diffusion [44]. Taking the study further, Van den Eeckhout et al. dissected the permeability shifts in post-cold and heat-treated specimens. Their findings revealed a dip in permeability post-cold treatment, which saw an uptick following heat treatment. This flux, they hypothesized, likely stemmed from heat treatment’s ability to purge certain point defects and instigate a reconfiguration of dislocation layouts [96].

## 6. Proposed Mechanisms of Hydrogen Embrittlement

### 6.1. Hydrogen-Enhanced Decohesion Mechanism (HEDE)

**Figure 10:**
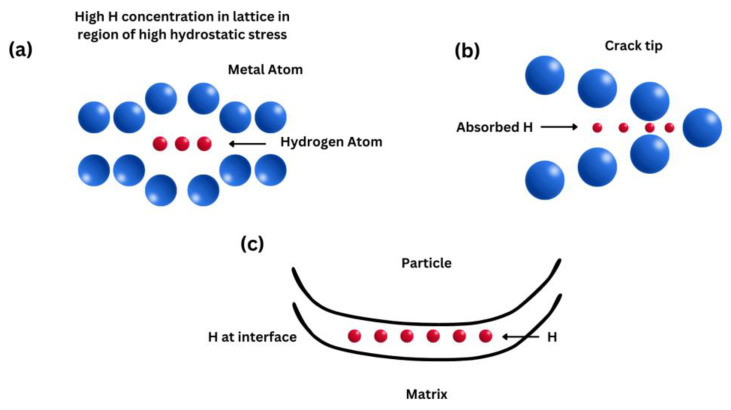
Schematic diagrams showing the HEDE mechanism, including tensile separation of atoms due to weakening of interatomic bonds by (**a**) hydrogen in the lattice, (**b**) hydrogen adsorbed at crack tips, and (**c**) hydrogen at particle–matrix interfaces.

The H-enhanced decohesion mechanism was first introduced in 1926 by Pfeil et al. [97], and it is a simple mechanism. They proposed that hydrogen decreases the cohesive strength across lattice planes and grain boundaries. Troiano in 1959 proposed that [98] the increasing interatomic repulsive forces and thus the decreasing atomic bond strength were due to the fact that the 1 s electron from the hydrogen tends to enter the unfilled 3 d shell of the iron atoms. However, apart from a few elements like Pd, the hydrogen solubility in metals is too low to cause a significant decohesion effect; in that case, hydrogen atoms are homogenously distributed in the microstructure [99,100]. Hence, a sufficiently high concentration of hydrogen needs to be accumulated for decohesion to occur. It has been proposed that high hydrogen concentrations can occur due to high hydrostatic stresses including strain gradient hardening [101]. A variety of locations for decohesion have been suggested [37,102]: (1) adsorbed hydrogen atoms at crack tips, (2) dislocation shielding regions at crack tips, (3) grain boundaries and interphase boundaries at crack tips, (4) sites of maximum hydrostatic stresses, and (5) particle/matrix interfaces (Figure 10).

Decohesion happens when the critical crack tip opening displacement (CTOD) is reached [103,104,105]. When hydrogen atoms are present in the microstructure and stresses are applied, then hydrogen atoms diffuse into the lattice structure and result in a reduction in cohesive strength at the crack tip and brittle cleavage-like fracture occurs. The surface energy of a material is decreased by reducing its cohesive strength so that fracture stress is also decreased and brittle fracture occurs below its allowable stress values. A major difficulty in proving this model is measuring the cohesive forces [104,106].

### 6.2. Hydrogen Pressure Theory

Zapffe et al. [107] presented a hydrogen pressure theory in 1941 suggesting that hydrogen atoms preferentially segregate at defect positions in the materials, such as micropores and inclusions. Then, locally accumulated hydrogen atoms gather to form hydrogen molecules. A high internal pressure is generated by the increase in hydrogen molecules. When the stress generated by the hydrogen gas pressure exceeds the yield strength of the material, hydrogen-induced cracking occurs. The concept of irreversible hydrogen embrittlement can be well explained by the hydrogen pressure theory.

### 6.3. Hydrogen-Enhanced Localized Plasticity (HELP)

**Figure 11:**
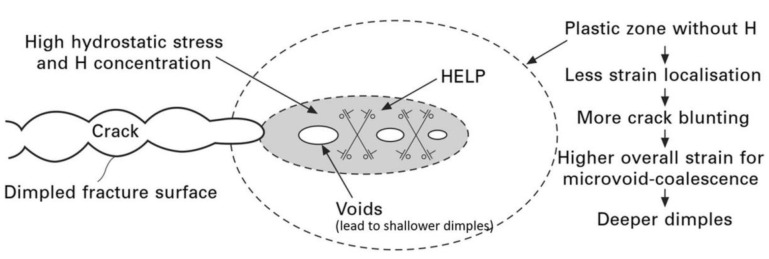
Schematic diagram illustrating the HELP mechanism [100].

This model was first suggested by Beachem [108] in 1972 and it is the most widely accepted mechanism. In this mechanism, hydrogen atoms accumulate near a crack tip. It also decreases the resistance to dislocation motion, increasing the mobility of dislocations. Therefore, dislocations act as carriers of plastic deformation in a metal lattice [106,109]. The presence of hydrogen around the dislocations results in a local drop in yield stress, and thus, a local movement of dislocations occurs at a low stress level (Figure 11). This implies that the fracture surfaces exhibit high localized plastic deformation near crack tips in embrittled materials and slip bands in those areas [110].

Large increases in dislocation mobility in the presence of hydrogen have also been observed by in situ transmission electron microscopy (TEM) observations [8,111,112,113,114]. Two reasons have commonly been postulated to cause this increased dislocation mobility. (1) Hydrogen reduces the repulsive interactions between dislocations and obstacles (e.g., secondary phases, solute atoms, and other dislocations) by creating a shielding effect. This reduction in interaction energy increases the mobility and slip positioning of dislocations and decreases the stress value required for local plastic deformation. The hydrogen-induced shielding effect applies more to edge dislocations than screw dislocations. (2) Hydrogen can reduce the yield strength of the material. This phenomenon is called the “softening effect”. The influence of hydrogen on the reduction in yield strength depends on the material, its purity, strain rate, temperature, and other factors [37,103,114,115]. For example, the degree of hydrogen-induced softening is sometimes large at low temperatures and low strain rates for pure iron single crystals, but is usually quite small for aluminum and nickel.

Nonetheless, this mechanism is also challenged by some experimental observations. For instance, tensile test results confirm that dislocations in IN718 alloys and pure aluminum are dragged by hydrogen [116]. In addition, it has been suggested that hydrogen impedes dislocation mobility according to simulation results [110,117]. Hence, it has commonly been assumed that the HELP system needs to combine with other systems to ultimately deteriorate material performance under a hydrogen atmosphere [118].

### 6.4. Adsorption-Induced Dislocation Emission (AIDE)

**Figure 12:**
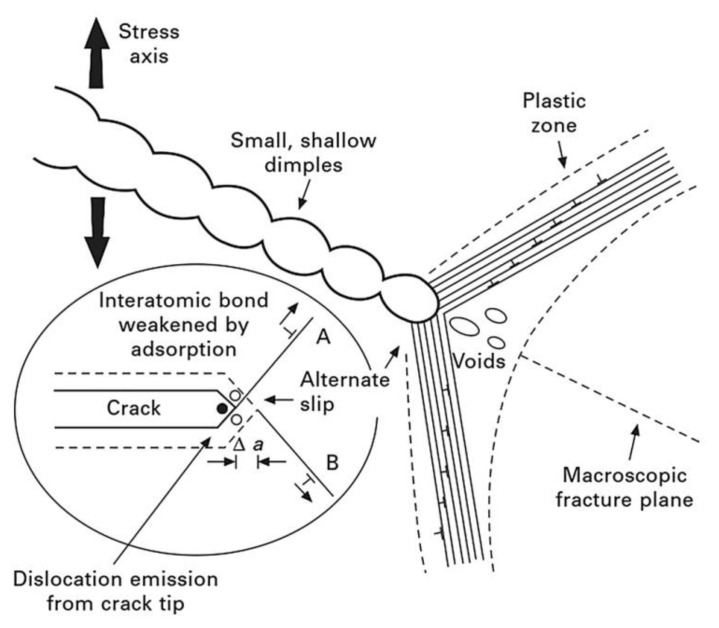
Schematic diagram illustrating the AIDE mechanism [100].

The adsorption-induced dislocation emission (AIDE) model was first proposed by Lynch [119] in 1976 and is a combination of both HEDE and HELP. In this model, the hydrogen atoms are adsorbed adjacent to a stress concentration area such as crack tips. The adsorption of hydrogen at crack tips weakens the interatomic bond energy and cohesive strength of materials through the HEDE mechanism and facilitates the subsequent emission of dislocations, then crack propagation by a slip step, and the generation of microvoids through the HELP mechanism [104,106,109,120]. The AIDE mechanism involves decohesion and dislocation injection from a crack tip facilitated by hydrogen adsorption, leading to nucleation and the growth of cracks (Figure 12). The formation of a slip step at the crack tip combined with microvoid coalescence results in crack propagation and fracture.

### 6.5. Hydride Formation

**Figure 13:**
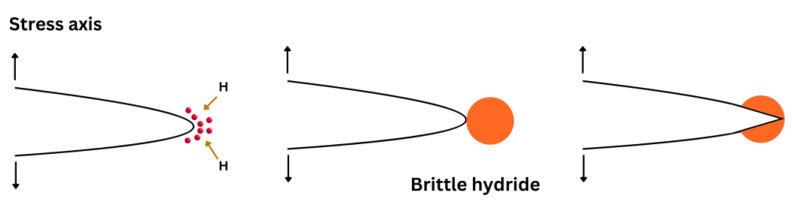
Schematic diagram showing subcritical crack growth including hydrogen diffusion to hydrostatically stressed regions, then formation and fracture of a brittle hydride at a crack tip.

Westlake (in 1969) [121] was the first to suggest a mechanism based on the formation and fracture of brittle hydrides at crack tips. Hydrides are generally responsible for cleavage fractures in specific materials such as Zr, V, Nb, Ti, and Ta [122,123]. The combination of these materials with hydrogen enables the formation of brittle hydrides because of their large bond energies. This mechanism consists of four stages: (1) hydrogen diffusion to crack tips, (2) formation and growth of a hydride phase, (3) cracking the hydride along a specific cleavage plane when it reaches a critical size, and (4) crack arrest at the matrix/hydride interfaces (Figure 13). As a result, crack propagation occurs through the repetition of the above sequence.

The hydrides can be divided into thermodynamically stable hydrides and stress-induced hydrides, considering the hydrogen concentration of the alloys. At high hydrogen concentrations, specific metals and their alloys can combine with hydrogen to form thermodynamically stable hydrides in the absence of stress. For stress-induced hydrides, a sufficiently high applied stress can act to redistribute the initial low hydrogen concentration. In these systems, hydrides are formed when the local hydrogen concentration reaches the solubility limit of the materials.

### 6.6. Hydrogen-Induced Reduction in Surface Energy

This theory was proposed by Uhlig [124] in 1967 based on the Griffith criterion for fracture in ideally brittle solids. This theory assumes that the adsorption of hydrogen reduces the surface energy and thus decreases the force needed to form new crack surfaces, and that the existence of a crack occurs where the hydrogen is adsorbed. The crack can more easily grow under lower mechanical load because of this decrease. Nevertheless, it is noteworthy that the magnitude of the reduction in the surface energy by hydrogen is quite small (e.g., 7% in the case of ferrite and 9% for austenite [125]), and considering this phenomenon, along with the plastic work of separation, renders the overall effect negligible [126].

### 6.7. Hydrogen-Enhanced Macroscopic Plasticity (HEMP)

This mechanism is also called hydrogen-enhanced macroscopic ductility and is related to the decrease in the yield strength due to hydrogen, attributed to solid solution softening by hydrogen atoms. It is certain that the beginning of yielding is accompanied by the movement of a significant number of dislocations. Therefore, the reduction in yield strength due to hydrogen indicates the easier macroscopic motion of significant dislocation masses facilitated by the presence of hydrogen. HEMP is quite different from the subcritical cracking mechanism of HELP. This is because there is no subcritical crack propagation involved in the reduction in the yield strength, and also, the plastic deformation is not localized but rather uniform throughout the whole gauge section [127].

### 6.8. Hydrogen Assisted Microvoid Coalescence

Microvoid coalescence is primarily a ductile fracture system and is attributed to the preferential trapping influences of microstructural heterogeneities on hydrogen atoms in front of the crack tip. Crack generation and growth happens in different stages such as void nucleation, void growth, void coalescence, and extension of the crack and eventual breaking of remaining existing ligaments by shear stress [104,128]. Due to the hydrogen impact, dislocation and localized plastic deformation take place in the material. Due to the joining of voids present in the crack growth direction, crack propagation takes place in a zig-zag pattern.

**Figure 14:**
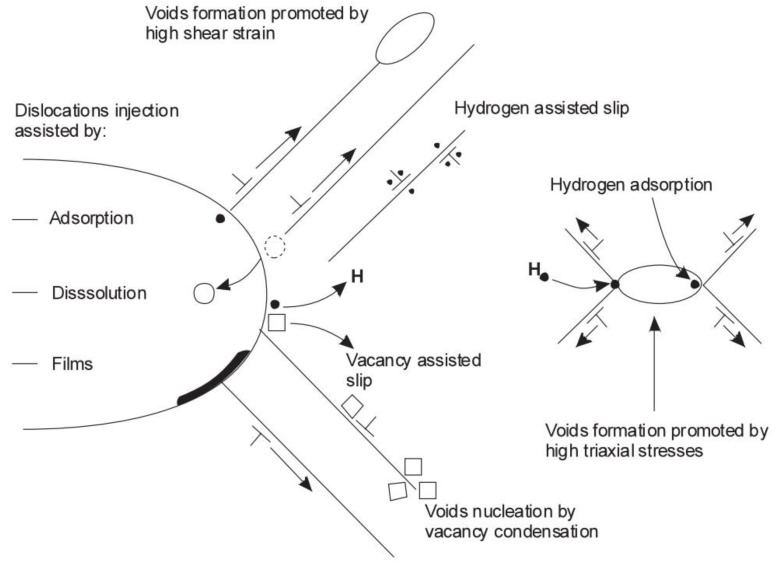
Processes resulting in hydrogen-induced cracking by localized slip and microvoid coalescence [129].

A summary of possible corrosion–deformation interactions which could lead to hydrogen-induced cracking is presented in Figure 14.

## 7. Hydrogen Embrittlement Characterization Techniques

To better understand the impact of hydrogen uptake in steels, it is necessary to establish experimental techniques to identify the presence and effect of hydrogen on steel microstructures and to provide valuable insights into the extent of its influence on the mechanical properties of the steels [130]. Microscopic observation, hydrogen permeation tests, thermal desorption analysis (TDA), and mechanical testing are the major categories of applied techniques in previous research [131]. Whilst the former three mainly aim to characterize the hydrogen content and its effect on microstructures, the latter aims to evaluate the influence of hydrogen adsorption on the mechanical properties of steels. The purpose of this section is to introduce mechanical testing methods, both established and new, that have provided valuable insights in HE research.

### 7.1. Tensile Tests

**Figure 15:**
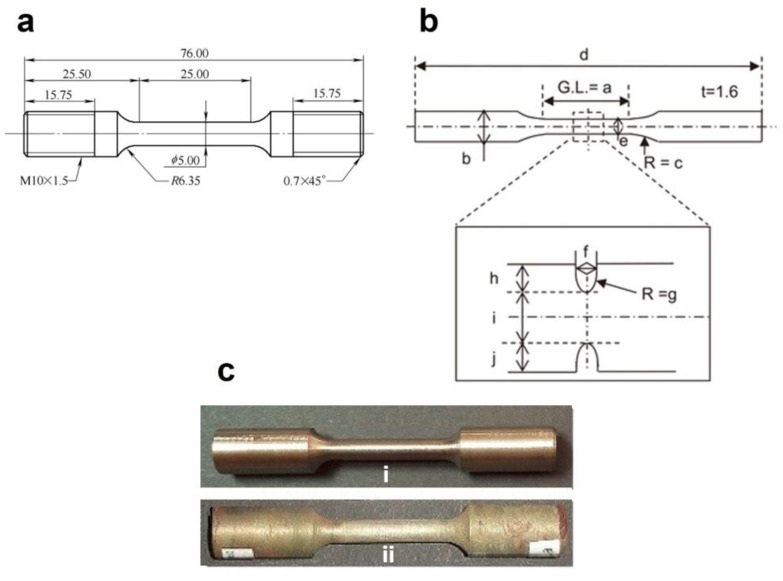
Tensile test specimens. (**a**) Typical drawing for smooth sample [138]. (**b**) Typical drawing for notched sample [136]. (**c**) Typical tensile specimen: (**i**) untreated and (**ii**) hydrogen embrittled [142].

Hydrogen-induced embrittlement may cause a loss of load-bearing capacity, leading to premature fracture and reduced ultimate tensile strength. Tensile testing of hydrogen-charged specimens allows for the determination of parameters such as the reduction in area and fracture surface morphology, providing insights into the fracture mode and the extent of hydrogen-induced damage [132,133,134]. Tensile tests may be conducted utilizing either pre-charged hydrogen (ex situ) or the introduction of hydrogen during the straining (in situ). Whilst the ex situ test is more widely adopted due to its simpler instrumentation involving a separate charging unit to the tensile test setup, the in situ approach is generally considered more representative (particularly for simulating steel pipe service conditions characterized by consistent and prolonged hydrogen pressure [135]), ensuring continuous hydrogen presence by integrating a hydrogen charging mechanism into the test setup [131]. The conventional strain rate test (CSRT) and slow strain rate test (SSRT) are the most commonly applied tensile tests in previous studies for investigating the HE susceptibility of steels conducted in both in situ and ex situ conditions. While the range of strain rate for the CSRT method is reported in the literature to be 1–12 mm/min [132,136,137], this range for the SSRT is recounted as 0.001–0.12 mm/min [136,138,139]. This controlled and gradual deformation rate better mimics the actual stress conditions experienced by materials in practical application, offering more reliable insights into a material’s response to hydrogen exposure and its potential for embrittlement [140]. The SSRT is standardized in ASTM G 129 [141]. Typical samples for tensile tests are shown in Figure 15a–c. Both smooth and notched geometries can be employed to differentiate the localized or universal effect of hydrogen-induced strain.

**Figure 16:**
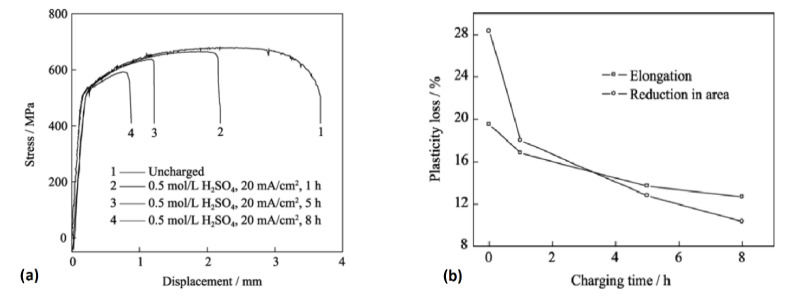
CSRT results for the X80 steel under different charging durations: (**a**) stress–displacement curves; (**b**) plasticity loss [132].

**Figure 17:**
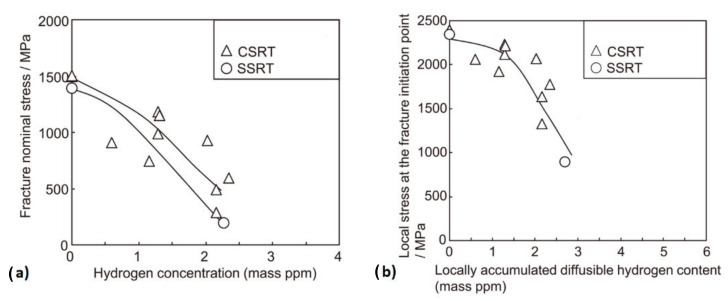
(**a**) Comparison of relationships between fracture nominal stress and hydrogen concentration of SCM435 steel obtained by CSRT and SSRT. (**b**) Comparison of relationships between local stress at fracture initiation points and local accumulated diffusible hydrogen concentration of SCM435 steel obtained by CSRT and SSRT [136].

The stress–displacement curves of X80 steel specimens after electrochemical hydrogen charging in a solution of 0.5 mol/L H_2_SO_4_ with a constant current density of 20 mA/cm^2^ and tensile testing by the CSRT method are shown in Figure 16a. This suggests that with increased charging time, a modest reduction in tensile strength became apparent at a limited scale. Furthermore, the assessment of hydrogen embrittlement sensitivity involves the utilization of relative plasticity damages, characterized by elongation loss rate (*I_δ_*) and area reduction rate (*I_ψ_*), calculated by the following formula and shown in Figure 16b for the above-mentioned specimen:

$$\left\{\begin{array}{ccc} I_{\delta }=\frac{\delta_{0}-\delta_{H}}{\delta_{0}}\times 100\% & \; & \left(27\right)\\ I_{\psi }=\frac{\psi_{0}-\psi_{H}}{\psi_{0}}\times 100\% & \; & \left(28\right) \end{array}\right.$$

where *δ*_0_, *δ_H_*, *ψ*_0,_ and *ψ_H_* are the elongation and reduction in area of the steel before and after hydrogen charging, respectively. Figure 16b indicates that as the duration of hydrogen charging increased, there was a tendency for the hydrogen content to rise, leading to a substantial decrease in the plasticity of the utilized X80 steel. Also, Takagi et al. [136] investigated the differences between the critical HE conditions of steels obtained by the CSRT and SSRT under a constant load condition for a 1300 MPa-class JIS-SCM435 steel as the representative material. Consequently, the assessment of hydrogen embrittlement’s critical conditions, as determined by applied stress and average diffusible hydrogen content (H_D_), followed a sequence aligned with the SSRT and CSRT, starting with low stress and hydrogen content levels (Figure 17a). A comparison of the critical conditions derived from these two techniques was also conducted using the fracture initiation point’s local stress and local diffusible hydrogen content index. It was observed that as the local diffusible hydrogen content increased, the local stress associated with critical conditions exhibited a decline (Figure 17b). In contrast, Hagihara [143,144] demonstrated that the critical condition of HE of TS 1300 MPa grade tempered martensitic steel obtained by the SSRT utilizing circumferential notched specimens yielded results nearly identical to the CSRT when assessing critical conditions based on local stress and local hydrogen distribution at the point of fracture initiation.

**Figure 18:**
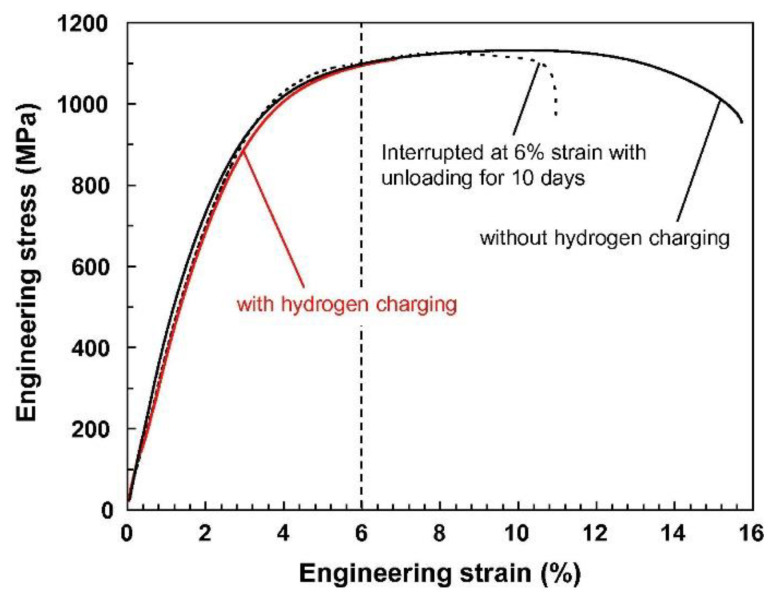
Engineered stress–strain curves of the specimens with and without charging and with interruption in the tensile test [145].

Koyama et al. [145] carried out an interrupted CSRT on a high-strength ferrite/martensite dual-phase (DP) steel and compared the results with the non-interrupted and without charging samples to investigate the involved HE mechanisms. During the interrupted test, hydrogen was introduced prior to conducting the tensile test, the test was halted when the strain reached 6%, and then the load was removed. Following a period of 10 days in contact with air, the sample was reloaded until it fractured. The contrast between results with and without hydrogen charging distinctly illustrates the HE effect. It is evident that the yield and tensile strengths remained largely unchanged following hydrogen charging, while the elongation before fracture experienced a significant decline. Importantly, the interrupted test showed a partial recovery in elongation due to the desorption of hydrogen, suggesting that the degradation of tensile properties caused by hydrogen was significantly influenced by both HEDE- and HELP-assisted crack propagation within the crack growth regime (Figure 18).

**Figure 19:**
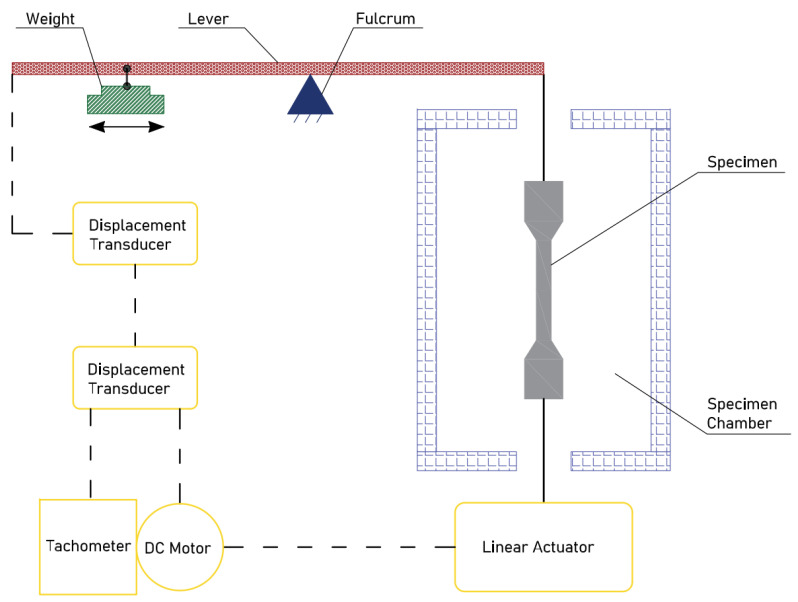
Schematic of the linear increasing stress testing (LIST) apparatus.

A drawback of the SSRT method was that upon surpassing the threshold stress (σ_TH_), the specimens underwent prolonged extension, leading to extended failure times and operational inconvenience. To address this challenge, a novel approach termed linearly increasing stress testing (LIST) was introduced [146,147,148]. This technique shares some similarities with the SSRT but offers distinct advantages. In the LIST method, a sample is subjected to a gradually increasing stress until failure occurs. This is accomplished through controlled weight displacement facilitated by a motor-driven mechanism, as illustrated in Figure 19. One important difference between the SSRT and LIST is that the SSRT operates based on displacement control, whereas LIST operates on load control [148]. It is reported that the LIST and SSRT are basically the same up to the initiation of the crack, yielding identical values for σ_TH_. The difference begins once the critical crack is reached. While it takes a relatively long time for the fracture of the SSRT sample to happen, the LIST sample fails at much shorter time due to experiencing plastic instability [149].

An earlier tensile testing method called the constant load test involves a notched or smooth specimen under an applied static load exposed to the environment (in situ). The constant load test was first introduced by Baldy [150] in the 1960s, and then became the NACE TM0177 [151] method A, also described in detail in ASTM E 1681 [152]. Typically, the assessment of HE susceptibility using this method relies on the time taken for failure to occur. Tensile test samples subjected to specific stress levels yield either a pass or fail outcome. By conducting tests on multiple specimens under different stress levels, it becomes possible to obtain an apparent threshold stress for HE [153]. Tensile tests can be performed either with constant-load or sustained-load (proof-ring or spring-loaded) devices as described in ASTM G49 [154]. While evaluating HE susceptibility through sustained-load test outcomes necessitates a visual inspection of specimens to identify crack presence, employing constant-load apparatus guarantees complete separation for materials prone to susceptibility. An issue frequently encountered in constant-load testing is the absence of a guarantee for sample failure, potentially leading to prolonged test durations. In such instances, a practical solution involves concluding the test after a specific duration (e.g., 100 h) has passed without the occurrence of specimen fracture [155].

**Figure 20:**
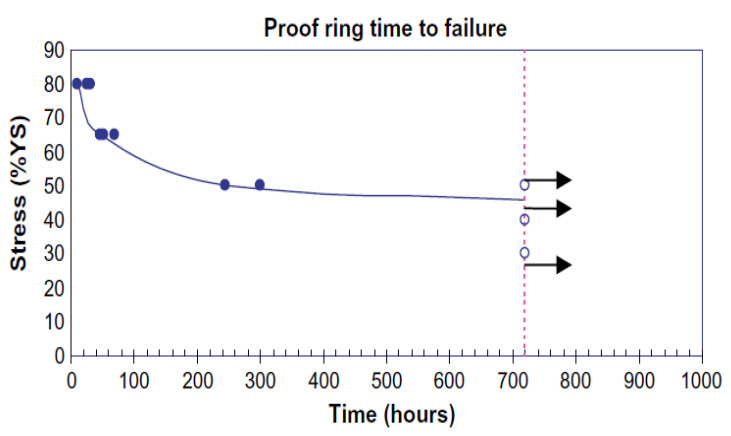
Proof-ring time to failure for the X100 in the NACE TM-0177 ‘‘A” solution (dark = fail, clear = pass; dashed line indicates the 720 h test duration) [156].

Al-Mansour et al. [156] investigated the HE susceptibility of API-X100 high-strength low-alloy steel using proof-ring constant-load testing with NACE TM-0177 solution A and generated an SCC threshold stress value of 46% of the yield strength. The specimens were loaded at stress values equivalent to 30% up to 80% of the material’s yield strength, and time to failure (TTF) or no failure was recorded based on a maximum test duration of 720 h. The results are shown in Figure 20. The low threshold stress value of the material was attributed mainly to the X100 microstructure, having a banded structure providing higher hydrogen trapping site density in front of the crack tip than homogenous microstructures [157]. Similar research has been conducted on other HSLA steels, X60, X65, and X70, reporting threshold stress values of 60%, 69%, and 80% of the YS, respectively [157,158,159], while quenching and tempering treatment has been shown to increase the threshold stress by removing the banded structure and provoking a more homogenous one [157].

**Figure 21:**
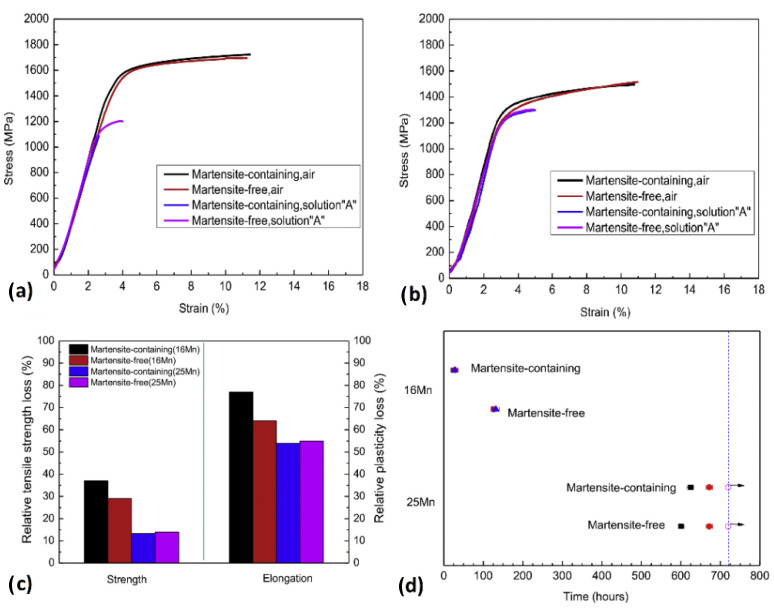
Influence of surface martensite layer on HE of 16Mn and 25Mn steels in different conditions in wet H_2_S environment: (**a**,**b**) engineered tensile stress–strain curves; (**c**) relative tensile strength and ductility loss; (**d**) proof-ring time to failure in the NACE TM0177 “A” solution [160].

Using the SSRT and proof-ring testing, Li et al. [160] investigated the influence of a surface martensite layer on the HE of TWIP (austenitic high-Mn twinning-induced plasticity) steels in a wet H_2_S environment. TWIP steels, owing to their mechanically induced austenitic twins and fcc structure of austenite phase, have an outstanding combination of both strength and ductility, and also high solubility and low diffusivity of hydrogen, making them good candidates for applications where high hardenability and formability is required at the same time as the high resistance to HE [161,162,163,164]. Two types of TWIP steels with different surface martensite microstructures were studied, Fe-16Mn-0.4C-2Mo (wt.%) (16Mn), with a surface layer containing ε-martensite, α’-martensite, and austenitic twins, and Fe-25Mn-0.4C-2Mo (wt.%) (25Mn), with a full α’-martensite surface layer. The results for the SSRT and proof-ring testing are shown in Figure 21a–d. It was seen that the strength reduction in 16Mn steel is approximately twice that of 25Mn steel due to the ε-martensite presence, which decreased hydrogen embrittlement resistance; removing surface martensite helped 16Mn steel but had little effect on 25Mn steel with only α’-martensite. The results from the proof-ring tests were also consistent with the tensile test.

### 7.2. Double Cantilever Beam Test

**Figure 22:**
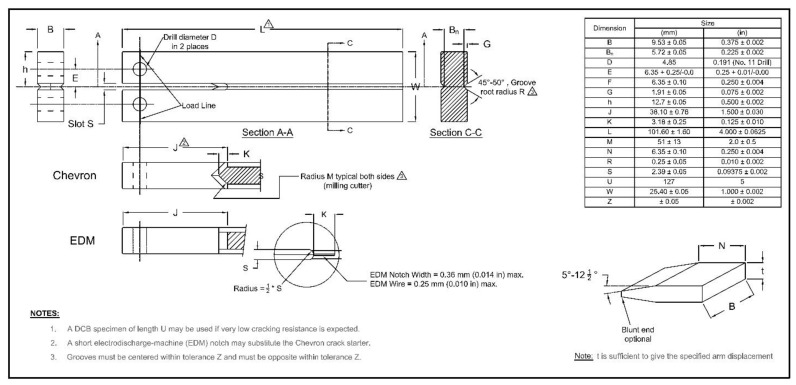
DCB test specimen.

The double cantilever beam (DCB) test is one of the most widely utilized tests to evaluate the resistance of steels to sulfide stress cracking (SSC), a particular form of HE. Due to its quantitative nature, high sensitivity, and minimal dependence on specimen surface finishing, the DCB test stands out as an exceptional quality control [165]. Initially proposed by Heady [166] in the early 1970s, the test is based on fracture mechanics, using the DCB specimen which is loaded to pre-define the critical stress intensity factor (*K_ISSC_*). Later, the test became the NACE TM0177 standard method D [163]. The standard DCB test specimen is shown in Figure 22 involving two beams (arms) separated by a slot. The specimen is then loaded either by inserting the wedge or by utilizing tensile equipment to produce an arm displacement which will create an initial stress intensity factor, *K_I0_*, at the chevron notch. In air, *K_I0_* is below *K_IC_*, so no crack propagation will occur; however, when the specimen is put in the sour environment (i.e., NACE standard solution), the steel becomes embrittled and the crack will grow, leading the specimen to be progressively unloaded. The crack growth eventually stops when the applied stress intensity factor, *K_I_*, matches the critical stress intensity factor of the steel in a corrosive environment, *K_ISSC_*. The value of *K_ISSC_* is calculated by the below equation:

(29) KISSC=Pa23+2.38haBBn13Bh3/2

where:

*K_ISSC_* = threshold stress intensity factor for SSC;

*P* = lift-off load;

*a* = crack length;

*h* = height of each arm;

*B* = DCB test specimen thickness;

*B_n_* = web thickness.

**Figure 23:**
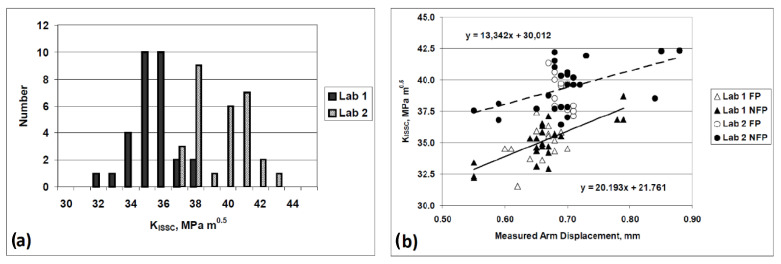
(**a**) Distribution of results by laboratory. (**b**) Arm displacement effect [175].

It is noteworthy to mention that the DCB test is only designed to compare the resistance of diverse steels to the HE and that the *K_ISSC_* is not an intrinsic material property [151], but also depends on test parameters such as specimen thickness and arm displacement [167,168,169,170]. In addition, the sensitivity of *K_ISSC_* to other factors has also been studied [171,172,173,174], and the following factors are considered to have a greater impact on the value of the calculated *K_ISSC_*: temperature, solution chemistry, specimen preparation, wedge introduction, etc. The test results for the two laboratories using the same material are compared by Szklarz [175] to investigate the effects of some of these factors. The test conditions were all the same in the two laboratories, except from the test vessel (12-liter capacity glass vessel in lab 1 and 6 or 10-liter depending on the number of tested specimens in lab 2), the use of a diffuser (lab 1 with diffuser and lab 2 without diffuser), and the opening of the specimens (with a hammer and chisel in lab 1 and with a tensile machine in lab 2). Figure 23a shows the results for the *K_ISSC_* values in both laboratories. It can be seen that there was around 12% difference between the results, which may be influenced in part by the actual arm displacement utilized by the two laboratories (shown on Figure 23b).

A similar trend for the effect of arm displacement on the K_ISSC_ value was reported by Linne et al. [176], Sponseller [165], and Asahi et al. [173]. Also, Moderer et al. [177] investigated the influence of arm displacement, initial crack length, pre-cracking, and notch type (slot with a chevron or an electro-discharged machine slot (EDM)) on the K_ISSC_ values. The results showed minor sensitivity of K_ISSC_ to the notch type and pre-cracking, but a higher number of valid specimens were attained for EDM-notched specimens. Furthermore, shorter initial crack length and higher arm displacement led to a slight increase in K_ISSC_ values.

**Figure 24:**
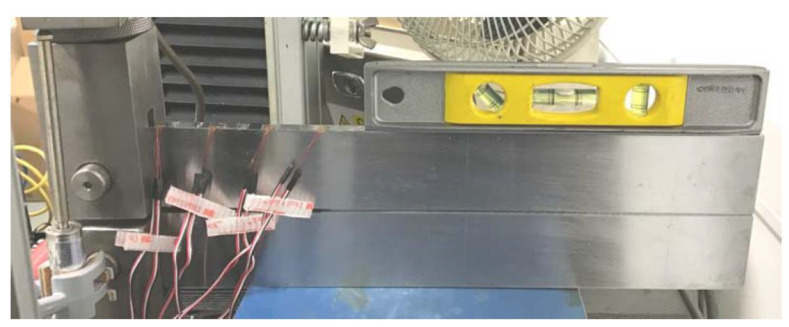
Large-scale DCB specimen from experiment by Maldonado [178] (the width and side notch dimensions were maintained within the NACE TM0177 limits, while all other dimensions were scaled up by a factor of 3.9x).

The DCB test is mainly designed to test the higher-strength materials in extreme sour environments, so there are limitations when applying this method to lower-strength steel grades (i.e., with SMYS values of ≤450 MPa) and mild sour environments due to crack growth beyond the acceptance criteria for a valid test and also the relaxation of stress at the crack tip because of arm bending. To overcome this issue, Maldonado et al. [178] conducted a large-scale DCB test (Figure 24) and obtained the K_ISSC_ value which met the requirement of the project.

**Figure 25:**
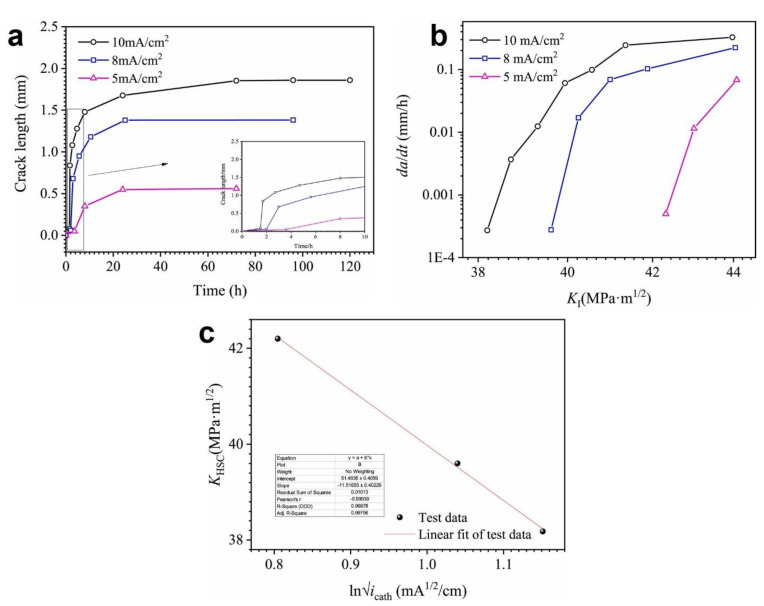
Hydrogen-induced cracking of X80 pipe steel under different current densities. (**a**) Variation curves of crack propagation length with time, (**b**) variation curves of crack propagation rate with stress intensity factor, and (**c**) relationship between K_HSC_ and subsurface absorbed hydrogen concentration C_0_ [181].

The stress field condition of the material plays a crucial role in influencing its hydrogen diffusion behavior and capture mechanism [179,180]. Xing et al. [181] investigated the relationship between the subsurface hydrogen content (C_0_) and the threshold stress intensity factor of the hydrogen-induced cracking arrest (K_HSC_) of X80 pipe steels through hydrogen permeation and DCB tests. For this purpose, samples were cut from the pipe steel and cathodically charged in a 0.5 mol/L H_2_SO_4_ and 0.2 g/L CH_4_N_2_S solution under different current densities, and crack propagation was monitored. As shown in Figure 25a, no crack propagation occurred when the current density was small (i.e., 1 or 3 mA/cm^2^), but for higher current densities, the crack length increased with the increase in applied current density. The computed threshold stress intensity factor K_HSC_ of hydrogen-induced crack diminished as the applied current density rose, as shown in Figure 25b. The values for the subsurface hydrogen concentration (C_0_) were also obtained from permeation tests with the same solution and current densities of the DCB test, and the relationship between C_0_ and K_HSC_ satisfied the expression of K_HSC_ α-lnC_0,_ as illustrated in Figure 25c.

### 7.3. Bent Beam Test

**Figure 26:**
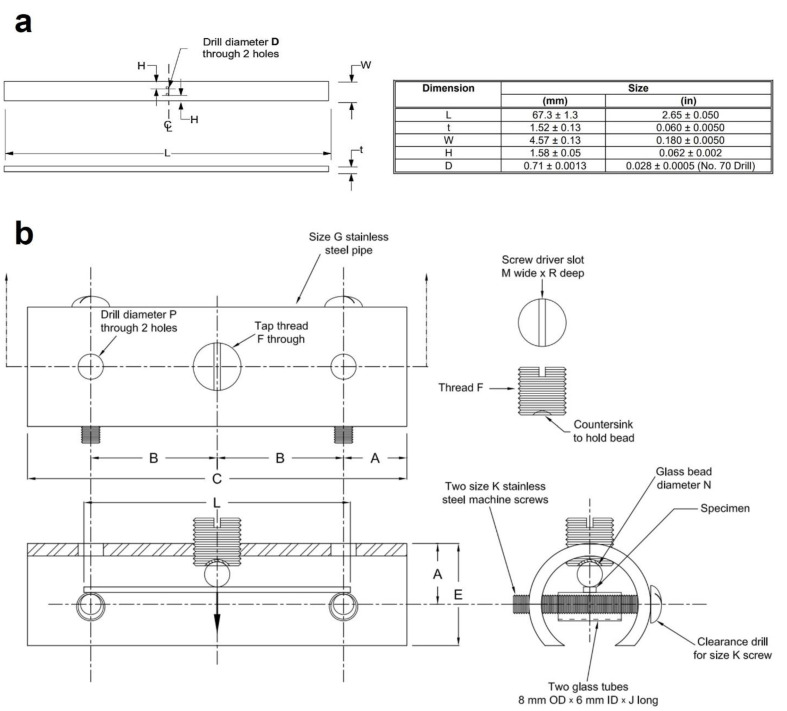
(**a**) Dimensional drawing of standard 3-point bent beam specimen. (**b**) Test fixture.

Bent beam tests are another kind of mechanical test utilized for the evaluation of HE susceptibility of carbon and low-alloy steels in the presence of a stress concentration, firstly introduced by Fraser [182] and later becoming the NACE method B [156]. Bent beam test specimens (Figure 26a) are loaded by test apparatus (Figure 26b) to varying particular deflections and then exposed to the test environment for a specific duration (i.e., 720 h), and failure/no failure of the test will be assessed based on observations of cracks in the specimens. A statistically based pseudo-stress (*S_c_*) for a 50% probability of failure is calculated to indicate the material’s resistance to SCC. For a three-point bending test (such as the NACE method B), the deflection of the test specimen is calculated by the below formula:

(30) $$D=\frac{Sl^{2}}{6Et}$$

where:

*D* = deflection;

*S* = nominal outer fiber pseudo-stress, typically in the range of 69 MPa from 22 to 24 HRC for carbon and low-alloy steel;

*l* = distance between centerlines of end supports;

*E* = elastic modulus;

*t* = thickness of test specimen.

Then, the pseudo-stress *S_c_* is calculated based on the below formula:

(31) $$S_{c}=\frac{\frac{\sum S}{68.95\;Mpa}+2\sum T}{n}$$

where:

*T* = the test result (i.e., +1 for passing and −1 for failure);

*n* = the total number of test specimens tested.

It should be noted that the computed pseudo-stress, lacking accuracy in reflecting the actual stress distribution, plastic deformation effects, and stress changes during crack growth, is unsuitable for determining threshold stress.

**Figure 27:**
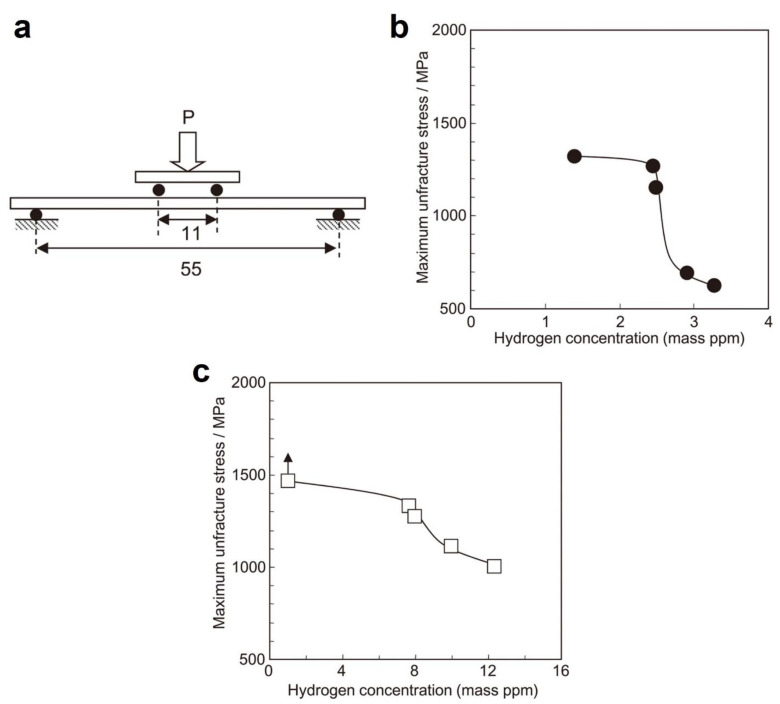
(**a**) Four-point bending specimen. (**b**,**c**) Diffusible hydrogen content–delayed fracture limit stress curves obtained by 4-point bending test for SCM435 and V-added ultra-high-strength steels, respectively [136].

Delayed fracture strength (DFS), which is the maximum bending stress that does not cause failure of the specimen, is another parameter for the evaluation of HE properties of materials obtained through four-point bend experiments [136,137,182,183]. In this method, the specimen (Figure 27a) is cathodically pre-charged with hydrogen, then loaded by four-point bending for a defined duration (i.e., 5 or 100 h), counting the fracture time from the start of the loading. The critical HE is established as the maximum applied load among conditions of the specimen that remain unfractured after the test duration. This method has more in common with proof-ring testing rather than the three-point bent beam test. The delayed fracture limit stress as a function of diffusible hydrogen content for two ultra-high-strength steels is shown in Figure 27b,c. In both steels, the DFS decreased with increasing H_D_; however, the V-added steel showed higher resistance to HE than the SCM435 steel at the same level of H_D_.

**Figure 28:**
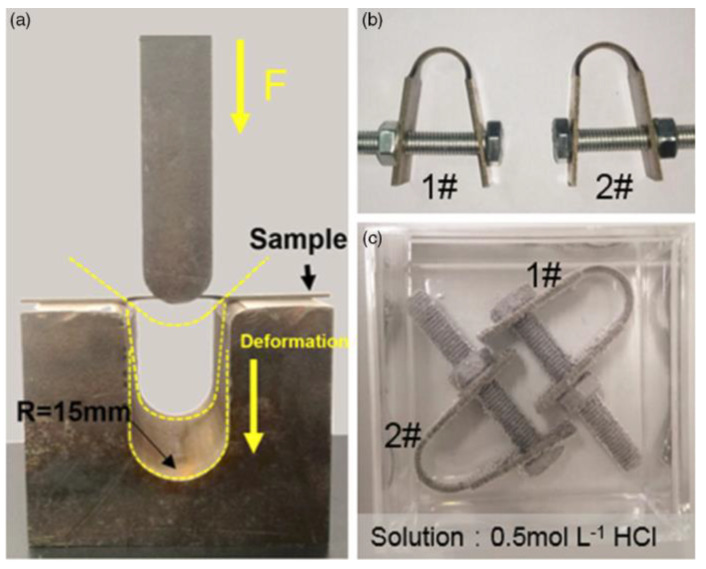
U-bend test: (**a**) test device, (**b**) sample pressed after forming, and (**c**) sample placed in 0.5 mol·L^−1^ HCl solution [184].

**Figure 29:**
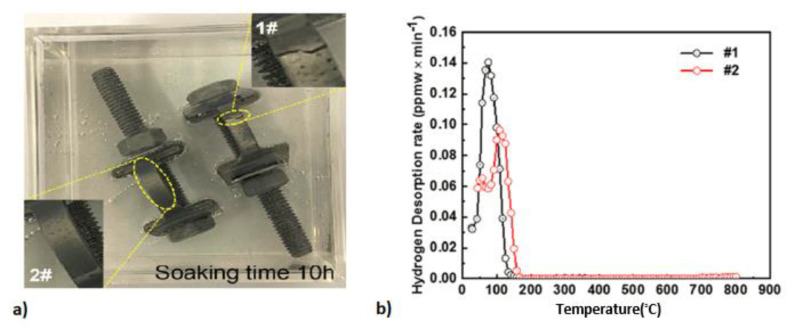
(**a**) Cracking of samples 1# and 2# after soaking for 10 h. (**b**) TDA spectra on #1 and #2 steels after hydrogen charging at a heating rate of 100 °C h^−1^ [184].

Si et al. [184] applied a U-bend test to compare the HE resistance of two 1500 MPa martensitic steels, in which a hydraulic press was used to impose constant pressure downward to form a 180° bend on the samples that were fixed by bolts. The deformed samples were then immersed in 0.5 mol·L^−1^ HCL solution and time to fracture was recorded to compare the HE resistance of the two samples (Figure 28a–c). While sample #1 had obvious cracking after 10 h of the bending immersion test (Figure 29a), sample #2 cracked after 50 h, indicating that #2 had a better anti-HE effect than #1 under equivalent conditions, which was attributed to their different microstructures and precipitated phases, leading to different values of hydrogen desorption rates (Figure 29b).

### 7.4. Fatigue Test

**Figure 30:**
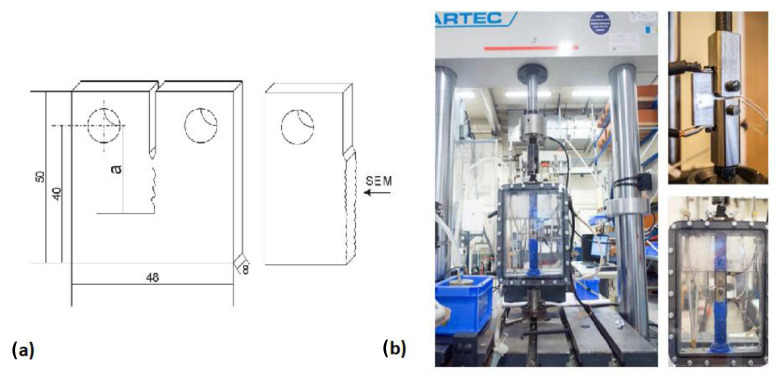
(**a**) Typical geometry of the CT specimen and schematic representation of the fracture surface. (**b**) Setup for in situ electrochemically charged fatigue crack growth rate test [16].

**Figure 31:**
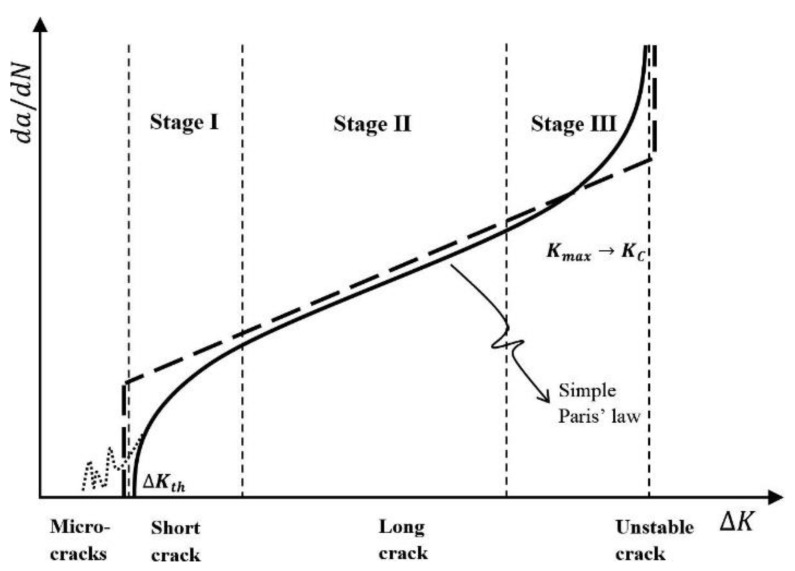
Schematic diagram of a normal fatigue cracking process [188].

Fatigue testing is another important method for characterizing hydrogen embrittlement. The presence of hydrogen significantly reduces the fatigue life of steels, making them more susceptible to fatigue crack initiation and propagation. Hydrogen-enhanced crack growth occurs due to the acceleration of crack growth rates under the influence of hydrogen atoms [15,185,186]. Fatigue testing involves subjecting hydrogen-charged specimens to cyclic loading, typically using techniques such as rotating beam or axial fatigue tests. The resulting fatigue life curves and crack growth rates can be used to assess the influence of hydrogen on the material’s fatigue behavior. If a significant amount of hydrogen enters into the material, it can have an adverse impact on the material’s static fracture and fatigue properties. This could lead to an undesirable rise in the rate at which cracks develop within the material, a phenomenon commonly denoted as hydrogen-affected fatigue crack growth rate (HAFCGR) [16]. Fatigue crack growth rate (FCGR) testing is usually performed on compact tension (CT) specimens. The recommended dimensions for CT specimens are described in the ASTM E647 standard [187] (Figure 30a). FCGR tests can be performed both ex situ and in situ; however, the in situ test has the advantage of reflecting the real condition of the material under the working environment. A typical test setup for in situ FCGR testing is shown in Figure 30b. The typical crack growth rate behavior in materials is characterized by ΔK-da/dN plots, which identify three domains: stage I (threshold domain), stage II (linear or Paris domain), and stage III (final fracture), as shown in Figure 31. While stage III is linked to unstable crack growth and failure, both stage I and particularly stage II (Paris domain) can be influenced by hydrogen presence [188]. Determining hydrogen’s impact on crack growth is challenging due to its dependence on various factors like the material, load frequency, temperature, pressure, or cathodic potential. The Paris law provides a quantitative description of the stage II fatigue crack growth domain [189]:

(32) $$\frac{da}{dN}=C.\Delta K^{m}$$

where:

*a* = crack length;

*N* = number of the cycles;

Δ*K* = variation in the stress intensity factor encountered by the material throughout fatigue cycles;

*C* and *m* = constants that depend on the material and the testing conditions.

**Figure 32:**
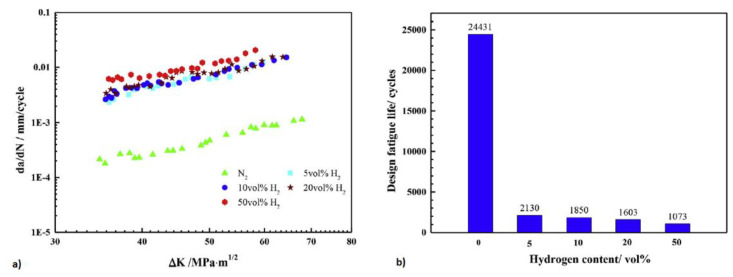
Hydrogen effects on X80 pipeline steel mechanical properties. (**a**) da/dN versus ΔK curves in nitrogen gas and hydrogen blends. (**b**) The fatigue life of the example pipeline [190].

Meng et al. [190] investigated the impact of hydrogen on mechanical properties of X-80 pipeline steel in natural gas/hydrogen mixtures with 5.0, 10.0, 20.0, and 50.0 vol% hydrogen at a pressure of 12 MPa using FCGR testing. As shown in Figure 32a,b, it was concluded that the quantity of introduced hydrogen is a crucial factor in the HE of X80 steel, and the rate of fatigue crack growth was notably accelerated as hydrogen levels increased. Also, the fatigue lifespan of the X80 steel pipeline experienced a significant decline due to the introduction of hydrogen. In a nitrogen gas environment, the fatigue life was 24,431 cycles, whereas in a 5% hydrogen blend, it reduced to 2130 cycles.

## 8. Conclusions

The interaction between hydrogen and metals is a highly intricate issue. This problem encompasses two aspects: firstly, the interaction of hydrogen with the metal surface, and secondly, the diffusion of hydrogen once it has entered the metal. Based on the aforementioned content, we can draw the following conclusions:

1. The interaction of hydrogen with metals involves three steps: physisorption, chemisorption, and diffusion into the steel.
2. The diffusion of hydrogen into metals is influenced by various factors. The impact of hydrogen traps is particularly significant. Point defects, line defects, plane defects, and volumetric defects in metal–hydrogen can all serve as hydrogen traps. These traps can be classified as reversible and irreversible based on their binding energy, but a case-by-case analysis is also pertinent. For instance, some traps might transition from being irreversible hydrogen traps at room temperature to reversible traps at elevated temperatures due to the temperature dependency of trap binding energy. Notably, though grain boundaries can expedite hydrogen diffusion due to the short-circuit effect, an excessively high density of grain boundaries can decrease the rate of hydrogen diffusion, as nodal points can capture hydrogen. Additionally, hydrogen can promote the clustering of vacancies in metals, leading to the formation of voids. As a consequence, molecular hydrogen can develop within these voids. This could result in severe degradation of the metal, which should be avoided.
3. The microstructure of metals also significantly influences hydrogen diffusion. For instance, cementite is considered one of the microstructures that enables rapid hydrogen diffusion. Apart from microstructures, the influence of phase boundaries is also pivotal, like the martensite/ferrite, martensite/austenite, and ferrite/austenite phase boundaries which act as reversible hydrogen traps.
4. It is evident that studying the behavior of hydrogen diffusion in metals should take into consideration all potential influencing factors. Therefore, a practical approach to understanding the impact of hydrogen traps on hydrogen permeation involves conducting specific permeation experiments for a given material and focusing on measuring and characterizing various types of hydrogen traps rather than concentrating on a single influencing factor. Moreover, the limitations posed by the present characterization methods demand the development of more innovative test techniques. Such a comprehensive investigation offers a more holistic perspective on hydrogen diffusion mechanisms and is instrumental in devising effective strategies to address and mitigate hydrogen-induced problems in materials.

## Nomenclature

**List of Symbols**	**Definition**
HE	hydrogen embrittlement
HIC	hydrogen-induced cracking
physisorption	physical adsorption
chemisorption	chemical adsorption
fcc	face-centered cubic
hcp	hexagonal close-packed
bcc	body-centered cubic
O sites	octahedral sites
T sites	tetrahedral sites
HMT	hydrogen microprint technique
SEM	scanning electron microscopy
TEM	transmission electron microscopy
AgBr	silver bromide
$NaNO_{2}$	sodium nitrite
$Na_{2}S_{2}O_{3}$	sodium thiosulfate
$NH_{4}SCN$	ammonium thiocyanate
$CH_{4}N_{2}S$	thiourea
$CH_{3}COOH$	acetic acid
$CH_{3}COONa$	sodium acetate
PSU	power supply unit
Pt	platinum
SSRT	slow strain rate tension test
CGHAZ	coarse-grained heat-affected zone
J	diffusion flux
D	diffusion coefficient
∂c/∂x	concentration gradient
∂c/∂t	the change in concentration over time
$\partial^{2}c/\partial x^{2}\;$	the second derivative of the concentration with respect to the distance
$c_{0R}$ ($\mathrm{mol}\cdot m^{-3}$)	the hydrogen concentration on the entry side (also referred to as charging side) in the hydrogen permeation test
$i_{t}$ (μA)	transient current in the hydrogen permeation test
$i_{\mathrm{ss}}$ (μA)	the steady-state current in the hydrogen permeation test
$t_{lag}$ (s)	the time elapsed from the beginning of the permeation test until the ratio of transient-to-steady-state current $\left(i_{t}/i_{\mathrm{ss}}\right)$ reaches 0.63
$t_{b}$ (s)	the time calculated by extrapolating the linear portion of the rising hydrogen permeation current transient
$D_{eff}$ ($m^{2}\cdot s^{-1}$)	the effective diffusion coefficient in the hydrogen permeation test
$J_{SS}$ ($\mathrm{mol}\cdot m^{-2}s^{-1}$)	permeation flux at steady state in the hydrogen permeation test
$i_{0}$ (μA)	background current in the hydrogen permeation test
$N_{T}$ ($m^{-3}$)	total density of hydrogen trap
$D_{l}$ ($m^{2}\cdot s^{-1}A$)	lattice diffusion coefficient of hydrogen
$E_{b}$ ($J\cdot {\mathrm{mol}}^{-1}$)	binding energy
$N_{L}$ ($m^{-3}$)	the density of the interstitial sites in the steel
$N_{ir}$ ($m^{-3}$)	the density of irreversible traps in the steel
$N_{r}$ ($m^{-3}$)	the density of reversible traps in the steel
ΔH ($J\cdot {\mathrm{mol}}^{-1}$)	dissolution enthalpy of hydrogen into steel
$f_{H_{2}}$ (MPa)	fugacity of hydrogen
LP	laser peening
HEDE	hydrogen-enhanced decohesion mechanism
CTOD	critical crack tip opening displacement
HELP	hydrogen-enhanced localized plasticity
AIDE	adsorption-induced dislocation emission
HEMP	hydrogen-enhanced macroscopic plasticity
TDA	thermal desorption analysis
CSRT	conventional strain rate test
$I_{\delta }$	elongation loss rate
$I_{\psi }$	area reduction rate
$\delta_{0}$	elongation before charging
$\delta_{H}$	elongation after charging
$\psi_{0}$	area reduction before charging
$\psi_{H}$	area reduction after charging
$\sigma_{TH}$	threshold stress
LIST	linearly increasing stress testing
SCC	stress corrosion cracking
TWIP	twinning-induced plasticity
DCB	double cantilever beam
SSC	sulfide stress cracking
$K_{ISSC}$	threshold stress intensity factor for SSC
$K_{I0}$	initial stress intensity factor
$K_{IC}$	critical stress intensity factor
a	DCB and fatigue test—specimen crack length
P	DCB test—lift-off load
h	DCB test—specimen height of each arm
B	DCB test—specimen thickness
B_n_	DCB test—specimen web thickness
EDM	electro-discharged machine
$H_{2}SO_{4}$	sulfuric acid
D	bent beam test—deflection
S	bent beam test—nominal outer fiber pseudo-stress
l	bent beam test—distance between centerlines of end supports
E	elastic modulus
t	bent beam test—thickness of test specimen
T	bent beam test result
n	bent beam test—total number of specimens tested
DFS	delayed fracture strength
HCl	hydrochloric acid
FGCR	fatigue crack growth rate
HAFCGR	hydrogen-affected fatigue crack growth rate
CT	compact tension
N	fatigue test—number of the cycles
ΔK	fatigue test variation in the stress intensity factor
